# Racial and Ethnic Disparities in Age-Specific All-Cause Mortality During the COVID-19 Pandemic

**DOI:** 10.1001/jamanetworkopen.2024.38918

**Published:** 2024-10-11

**Authors:** Jeremy Samuel Faust, Benjamin Renton, Tasce Bongiovanni, Alexander Junxiang Chen, Karen Dorsey Sheares, Chengan Du, Utibe R. Essien, Elena Fuentes-Afflick, Trent Haywood, Rohan Khera, Terris King, Shu-Xia Li, Zhenqiu Lin, Yuan Lu, Andrew D. A. Marshall, Chima D. Ndumele, Ijeoma Opara, Tina Loarte-Rodriguez, Mitsuaki Sawano, Kekoa Taparra, Herman A. Taylor, Karol E. Watson, Clyde W. Yancy, Harlan M. Krumholz

**Affiliations:** 1 Department of Emergency Medicine, Mass General Brigham, Boston, Massachusetts; 2 Division of Health Services Research, Harvard Medical School, Boston, Massachusetts; 3 Ontos Analytics, Cambridge, Massachusetts; 4 Brown University School of Public Health, Cambridge, Massachusetts; 5 University of California, San Francisco School of Medicine, San Francisco; 6 Department of Pediatrics, Yale School of Medicine, New Haven, Connecticut; 7 Center for Outcomes Research and Evaluation, Yale New Haven Hospital, New Haven, Connecticut; 8 Yale New Haven Hospital Center for Outcomes Research and Evaluation, New Haven, Connecticut; 9 Division of General Internal Medicine and Health Services Research, David Geffen School of Medicine at UCLA, Los Angeles, California; 10 Knowality, LLC, Chicago, Illinois; 11 Section of Cardiovascular Medicine, Department of Internal Medicine, Yale School of Medicine, New Haven, Connecticut; 12 The Grace Foundation, Baltimore, Maryland; 13 Department of Health Policy and Management, Yale School of Public Health, New Haven, Connecticut; 14 Yale University School of Public Health; 15 Stanford University, Stanford, California; 16 Morehouse School of Medicine, Atlanta, Georgia; 17 Division of Cardiology, David Geffen School of Medicine at UCLA, Los Angeles, California

## Abstract

**Importance:**

The end of the COVID-19 public health emergency (PHE) provides an opportunity to fully describe pandemic-associated racial and ethnic mortality disparities. Age-specific excess mortality differences have important downstream implications, especially in minoritized race and ethnicity populations.

**Objectives:**

To characterize overall and age-specific all-cause excess mortality by race and ethnicity during the COVID-19 PHE and assess whether measured differences reflected changes from prepandemic disparities.

**Design, Setting, and Participants:**

This cross-sectional study analyzed data of all US residents and decedents during the COVID-19 PHE, aggregated by observed race and ethnicity (at time of death) and age. Statistical analysis was performed from March 2020 to May 2023.

**Exposures:**

COVID-19 PHE period (March 2020 to May 2023).

**Main Outcomes and Measures:**

All-cause excess mortality (incident rates, observed-to-expected ratios) and all-cause mortality relative risks before and during the PHE.

**Results:**

For the COVID-19 PHE period, data for 10 643 433 death certificates were available; mean (SD) decedent age was 72.7 (17.9) years; 944 318 (8.9%) were Hispanic; 78 973 (0.7%) were non-Hispanic American Indian or Alaska Native; 288 680 (2.7%) were non-Hispanic Asian, 1 374 228 (12.9%) were non-Hispanic Black or African American, 52 905 (0.5%) were non-Hispanic more than 1 race, 15 135 (0.1%) were non-Hispanic Native Hawaiian or Other Pacific Islander, and 7 877 996 (74.1%) were non-Hispanic White. More than 1.38 million all-cause excess deaths (observed-to-expected ratio, 1.15 [95% CI, 1.12-1.18]) occurred, corresponding to approximately 23 million years of potential life lost (YPLL) during the pandemic. For the total population (all ages), the racial and ethnic groups with the highest observed-to-expected all-cause mortality ratios were the American Indian or Alaska Native (1.34 [95% CI, 1.31-1.37]) and Hispanic (1.31 [95% CI, 1.27-1.34]) populations. However, higher ratios were observed in the US population aged 25 to 64 years (1.20 [95% CI, 1.18-1.22]), greatest among the American Indian or Alaska Native (1.45 [95% CI, 1.42-1.48]), Hispanic (1.40 [95% CI, 1.38-1.42]), and Native Hawaiian or Other Pacific Islander (1.39 [95% CI, 1.34-1.44]) groups. In the total population aged younger than 25 years, the Black population accounted for 51.1% of excess mortality, despite representing 13.8% of the population. Had the rate of excess mortality observed among the White population been observed among the total population, more than 252 000 (18.3%) fewer excess deaths and more than 5.2 million (22.3%) fewer YPLL would have occurred.

**Conclusions and Relevance:**

In this cross-sectional study of the US population during the COVID-19 PHE, excess mortality occurred in all racial and ethnic groups, with disparities affecting several minoritized populations. The greatest relative increases occurred in populations aged 25 to 64 years. Documented differences deviated from prepandemic disparities.

## Key Points

**Question:**

What was the extent of racial and ethnic differences in all-cause excess mortality during the COVID-19 public health emergency (PHE) by age, and were disparities departures from prepandemic norms?

**Findings:**

This cross-sectional study measured more than 1.38 million PHE-associated all-cause excess deaths and found that in the overall population, the highest observed-to-expected mortality ratios occurred among non-Hispanic American Indian or Alaska Native and Hispanic populations. However, observed-to-expected mortality ratios were highest in the population aged 25 to 64 years, particularly among American Indian or Alaska Native, Hispanic, and Native Hawaiian or Other Pacific Islander groups; among people aged at least 25 years, the Black population accounted for 51.1% of excess mortality; disparities deviated from prepandemic baselines.

**Meaning:**

These results suggest that the COVID-19 PHE disproportionately affected several minoritized racial and ethnic groups; the largest relative increases occurred in the population aged 25 to 64 years, implying lasting downstream consequences.

## Teaser

This cross-sectional study describes overall and age-specific all-cause excess mortality by race and ethnicity during the COVID-19 public health emergency and assesses whether measured differences were associated with prepandemic disparities.

## Introduction

The end of the US COVID-19 public health emergency (PHE) was declared in May 2023. Measuring and analyzing the final excess mortality figures and disparities by race and ethnicity during the acute phase of the pandemic stands to yield powerful insights regarding shortcomings in our preparation and response. We aimed to characterize differing excess mortality by race and ethnicity and to determine if disparities differed by age, as excess deaths occurring in younger generations manifestly carry greater immediate and long-term effects—especially for populations affected by the various detriments of systemic discrimination and racism. We further sought to determine whether existing (prepandemic) racial and ethnic mortality disparities during the COVID-19 emergency were augmented by the pandemic, as opposed to replicated but at higher levels.

Therefore, we assessed mortality disparities by race and ethnicity during the COVID-19 pandemic, overall and stratified by age groupings, during the US COVID-19 PHE. Our focus on all-cause mortality allowed a comprehensive perspective of the pandemic’s overall association with outcomes, capturing direct COVID-19 deaths (including miscategorized COVID-19–related deaths) and indirect consequences of the pandemic, including for populations frequently excluded from public-facing reports and dashboards.^1,2,3,4,5,6,7,8^ Using Centers for Disease Control and Prevention (CDC) data, we modeled and measured cumulative all-cause excess mortality, analyzed the age distribution of excess deaths, and estimated the years of potential lost life (YPLL) for each race and ethnicity and for age groups, and determined how many deaths and YPLL could have been averted had subgroup observed-to-expected all-cause mortality ratios replicated those observed in the White population. To determine whether existing disparities were replicated or augmented during the pandemic, prepandemic and pandemic all-cause mortality risks by race and ethnicity and age were measured, with attention to differences during the prevaccine and vaccine eras.

## Methods

This cross-sectional study was conducted using deidentified, publicly available data and, as such, did not require institutional review board approval or informed patient consent, in accordance with 45 CFR §46.^9^ We followed the Strengthening the Reporting of Observational Studies in Epidemiology (STROBE) reporting guideline.^10^

We conducted a cross-sectional study of the US population’s exposure to the COVID-19 pandemic. To measure pandemic-associated mortality by race and ethnicity, we adapted established methods for computing age-adjusted rates, estimating excess mortality and YPLL.^1,2,3,4,5,6,7^ Race and ethnicity was determined based on observed CDC single race designations and/or dimensions, as reported on death certificates in CDC Wide-Ranging Online Data for Epidemiologic Research (WONDER), typically by funeral directors with input from next of kin (eMethods in Supplement 1).^8^ We accordingly divided the US population into 7 race and ethnicity groups: non-Hispanic American Indian or Alaska Native, non-Hispanic Asian (Asian), non-Hispanic-Black or African American (Black), Hispanic of all races (Hispanic), non-Hispanic Native Hawaiian or Other Pacific Islander, and non-Hispanic White (White). Persons who reported more than 1 race were included for US totals but were not analyzed as a group.^11^ Hispanic ethnicity was not stated for 0.0027% of deaths and these deaths were excluded (race categorization was available for all deaths). We divided each race and ethnicity into 3 age groups: younger than 25 years, 25 to 64 years, and 65 years or older. Therefore, the all-ages results for each group were summations of age-stratified models (rather than age adjustments and/or standardizations of the total populations). The study period was March 1, 2020, to April 30, 2023.

### Data

We used deidentified, publicly available death and US Census population data from the CDC WONDER. This data source encompasses all deaths among US residents.^12^

### Statistical Analysis

#### Excess Mortality

Excess mortality was defined as the number of raw observed deaths minus modeled expected deaths.^6,7^ To estimate expected deaths, we used seasonal, autoregressive integrated moving averages (ARIMA) for all-cause mortality by component demographic (race and ethnicity and age grouping). For each demographic, ARIMA models were trained using prepandemic population data (2014-2020) to estimate yearly populations for 2021 to 2023 (estimated yearly changes were divided evenly over 12 months). Seasonal ARIMA models were trained on monthly death counts (62 prepandemic months, January 2015-February 2020) for each age group, using the estimated monthly populations as covariates to overcome stationarity. Composite all-ages estimates were summed from the component age-group models and populations were corrected for cumulative excess mortality (eMethods in Supplement 1). Excess mortality was measured during the prevaccine pandemic era (starting March 2020) and during the vaccine era (starting March 2021 for ages 65 years and older, and May 2021 for ages 25 to 64 years).

#### Years of Potential Life Lost

To estimate total and per capita years of potential life lost (YPLL), we modeled excess deaths for each race and ethnicity by 10-year age group (except less than 25 years and greater than 85 years) and applied CDC life table figures to calculate the specific expectation of life for single year ages for each race and ethnicity (eMethods in Supplement 1).^13^

#### Potential Excess Deaths and YPLL Averted

Potential excess deaths and YPLL averted were calculated by applying the observed-to-expected ratios observed in the same-age White population to each race and ethnicity group and summed for overall tallies (eMethods in Supplement 1).^14,15^

#### Excess Mortality by Underlying Cause of Death *ICD-10* Chapter

An analysis of excess mortality by underlying cause of death *International Statistical Classification of Diseases and Related Health Problems, Tenth Revision (ICD-10)* chapter (eMethods in Supplement 1) was conducted to characterize disparities by specific medical (ie, natural) causes and external manner of death (eg, accidents, homicide). We applied the methods described above, with two modifications based on data availability (eMethods in Supplement 1). Spearman coefficients correlating (nonnormally distributed) all-cause excess mortality and select medical causes of death (including COVID-19; *ICD-10* U00-U99: Codes for special purposes, of which COVID-19 comprised >99.992% of deaths) and for external manner of death, were determined to assess temporal associations. For correlations strength ranges (eMethods in Supplement 1).^16^

#### Changes in Mortality Disparities

To determine whether prepandemic mortality disparities by race and ethnicity changed at the onset of the pandemic and over its course, we measured prepandemic (March 2015-February 2020) and pandemic relative risks (RR) for age-standardized all-cause mortality incidence rates for all groups, using contemporaneous same-age White population as comparators. Yearly and monthly RRs were determined for prepandemic (eg, March 2015-February 2016) and pandemic years (eg, March 2020-February 2021). The 95% CIs for all RRs were determined via the geometric means of monthly RRs within specific periods (yearly, prepandemic, and pandemic).

Analyses were performed in R 4.0.3 and Microsoft Excel 16.74 from March 2020 to May 2023. Exhibits were created in Flourish (Canva UK Operations, London, UK).

## Results

Among the 331.2 million US residents (pandemic mean), data for 10 643 433 death certificates were available at the time of analysis; mean (SD) decedent age was 72.7 (17.9) years; 944 318 (8.9%) were Hispanic; 78 973 (0.7%) were non-Hispanic American Indian or Alaska Native; 288 680 (2.7%) were non-Hispanic Asian, 1 374 228 (12.9%) were non-Hispanic Black or African American, 52 905 (0.5%) were non-Hispanic more than 1 race, 15 135 (0.1%) were non-Hispanic Native Hawaiian or Other Pacific Islander, and 7 877 996 (74.1%) were non-Hispanic White.

### Excess Deaths and Years of Potential Lost Life

**Figure 1.**
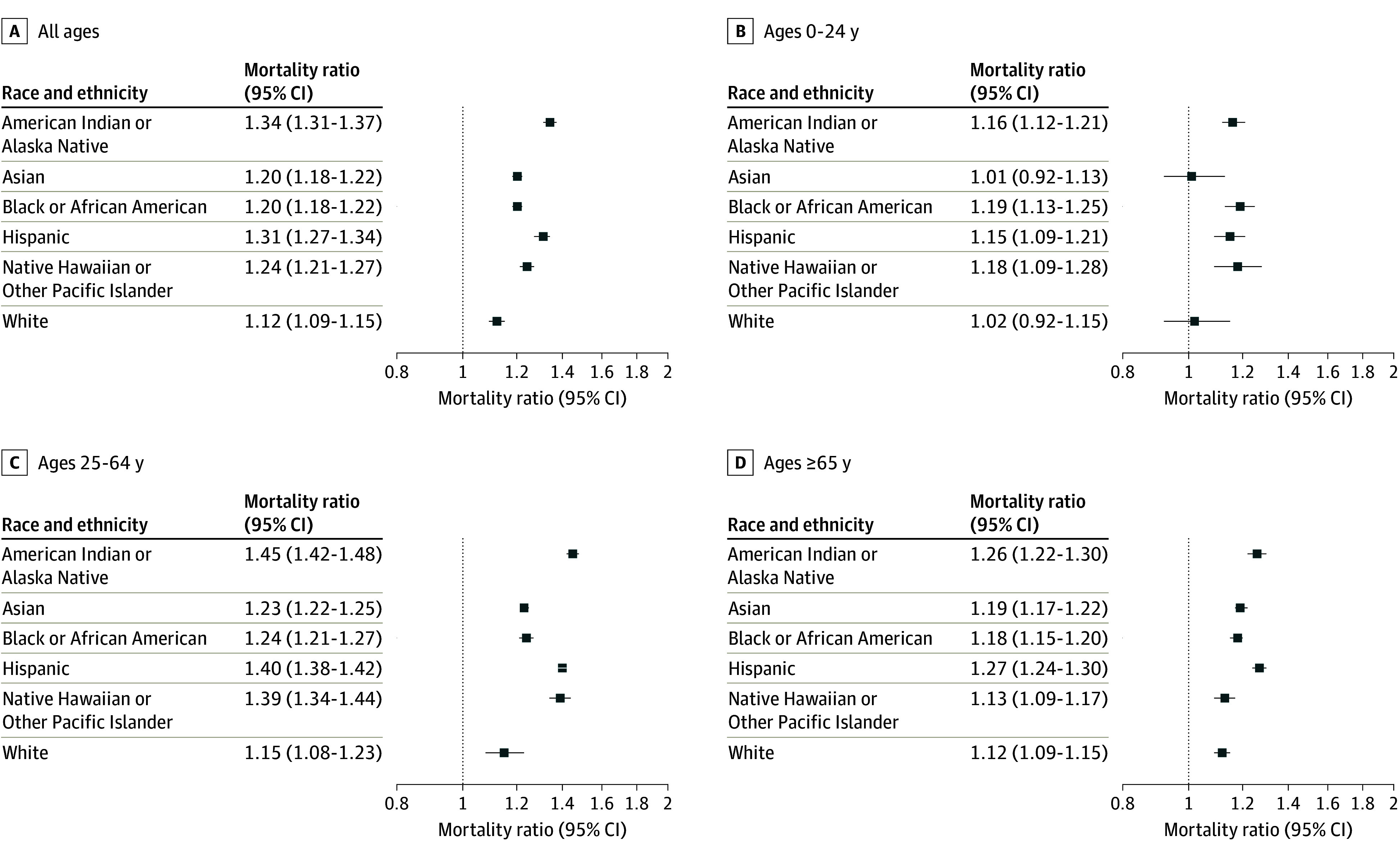
Observed-to-Expected Ratios for All-Cause Mortality During the Pandemic Period by Race and Ethnicity For each group, the graphed ratio reflects raw observed deaths divided by the number of modeled expected deaths during the pandemic period for that specific population; 95% CIs are shown as horizontal bars. The vertical line (1.0) means no change.

There were greater than 1.38 million all-cause excess deaths (observed-to-expected ratio, 1.15 [95% CI, 1.12-1.18]) and approximately 23 million corresponding years of potential life lost (YPLL) during the pandemic. Had the rate of excess mortality observed among the White population been observed among the total population, more than 252 000 (18.3%) fewer excess deaths, corresponding to approximately 5.2 million (22.3%) fewer YPLL would have occurred, including greater than 133 800 fewer excess deaths and greater than 2.9 million YPLL in the Hispanic population alone (Tables 1 and 2). Raw counts, incidence rates, and observed-to-expected ratio for mortality varied by race and ethnicity among all age groups (Table 1, Figure 1; eFigures 1-3 in Supplement 1). The highest observed-to-expected ratios were documented among the American Indian or Alaska Native (1.34 [95% CI, 1.31-1.37]) and Hispanic (1.31 [95% CI, 1.27-1.34]) populations (Figure 1A).

**Table 1.** All-Cause Mortality, Excess Deaths, and Potential Excess Deaths Averted, by Race and Ethnicity

Race and ethnicity	Expected deaths (95% CI)	Observed deaths	Excess deaths, No. (95% CI)	Excess deaths, IR (95% CI)	Ratio of observed to expected deaths (95% CI)	Potential deaths averted, No. (%)	<25 y share, %	25-64 y share, %	≥65 y share, %
American Indian or Alaska Native	59 203 (57 717-60 689)	79 214	20 011 (18 525-21 497)	822.25 (761.2-883.31)	1.34 (1.31-1.37)	12 529 (62.6)	2.20	58.30	39.50
Asian	241 391 (237 821-244 961)	289 372	47 981 (44 411-51 551)	246.15 (227.84-264.47)	1.20 (1.18-1.22)	18 823 (39.2)	0.20	24.00	75.90
Black or African American	1 149 070 (1 126 327-1 171 812)	1 377 386	228 316 (205 574-251 059)	549.11 (494.41-603.81)	1.20 (1.18-1.22)	85 703 (37.5)	3.90	42.60	53.40
Hispanic or Latino	723 093 (703 422-742 764)	945 200	222 107 (202 436-241 778)	355.1 (323.65-386.55)	1.31 (1.27-1.34)	133 815 (60.2)	2.70	43.90	53.40
More than 1 race	47 367 (45 171-49 562)	53 023	5656 (3461-7852)	72.47 (44.34-100.6)	1.12 (1.07-1.17)	130 (2.3)	0.00	60.20	39.80
Native Hawaiian or Other Pacific Islander	12 225 (11 892-12 557)	15 147	2922 (2590-3255)	470.21 (416.76-523.67)	1.24 (1.21-1.27)	1388 (47.5)	3.60	66.70	29.70
White	7 028 654 (6 843 894-7 213 414)	7 884 091	855 437 (670 677-1 040 197)	436.81 (342.47-531.15)	1.12 (1.09-1.15)	Reference	0.20	24.90	74.80
All races	9 261 002 (9 048 489-9 473 516)	10 643 433	1 382 431 (1 169 917-1 594 944)	418.52 (354.18-482.85)	1.15 (1.12-1.18)	252 389 (18.3)	1.30	31.60	67.10

**Table 2.** Years of YPLL and Potential YPLL Averted by Race and Ethnicity

Race and ethnicity	YPLL	Potential YPLL averted	<25 y YPLL	25-64 y YPLL	≥65 y YPLL	Population per YPLL	YPLL per excess death
American Indian or Alaska Native	405 716	268 796 (66.3)	23 774	308 062	73 880	6	20.8
Asian	758 762	281 746 (37.1)	5333	356 746	396 683	25.8	15.9
Black or African American	4 289 225	1 816 586 (42.4)	542 891	2 534 603	1 211 731	9.7	18.6
Hispanic or Latino	4 661 690	2 899 639 (62.2)	395 015	2 993 509	1 273 166	13.4	20.8
More than 1 race	135 194-186 408	9201 (4.9)	−6557	160 808	32 157	41.9-57.7	21.1-29.1
Native Hawaiian or other Pacific Islander	70 238-95 961	49 941 (52.0)	7548	73 263	15 150	6.5-8.8	21.4-29.2
White	12 899 721	Reference	136 959	6 743 271	6 019 491	15.2	14.4
All races	23 220 547	5 192 293 (22.3)	1 104 723	13 104 482	9 011 342	14.2	16.2

Abbreviation: YPPL, years of potential life lost.

Centers for Disease Control and Prevention life tables do not currently include Native Hawaiian or other Pacific Islander or more-than-1 race categories. Therefore, ranges are supplied based on applying life expectancy figures from the group with the lowest life expectancy (American Indian or Alaska Native) and the highest (Asian). Data for the more-than-1 race category are not shown but are included in the figures for all races (bottom row).

### Age-Specific Excess Deaths and Disparities

Age-specific excess mortality rates, observed-to-expected ratios, and other related figures are shown in Figure 1B-1D (eTables 1-3 in Supplement 1). Notable age-specific observations are included in the following sections.

#### Ages Younger Than 25 Years 

Excess deaths included approximately 9000 Black (approximately 542 000 YPLL), approximately 6000 Hispanic (approximately 395 000 YPLL), approximately 400 American Indian or Alaska Native (approximately 24 000 YPLL), and 100 Native Hawaiian and Other Pacific Islander people (approximately 7500 YPLL) (95% CIs for excess mortality and corresponding YPLL among younger Asian and White people crossed 0) (Figure 1B; eTable 1 in Supplement 1). The share of excess mortality exceeded the share of the population among American Indian or Alaska Native, Black, and Native Hawaiian or Other Pacific Islander populations (eTable 4 in Supplement 1, all ages and by age group). Notably, the Black population accounted for a majority (51.4%) of excess mortality despite representing only 13.8% of the age demographic population.

#### Ages 25 to 64 Years

The population aged 25 to 64 years experienced the highest relative increase in all-cause mortality (1.20 [95% CI, 1.18-1.22) of any age group studied, a finding which held in every race and ethnicity group (Figure 1B-1D; eTables 1-3 in Supplement 1), despite higher excess mortality incident rates in the population aged 65 years and older) (eTable 3 in Supplement 1). In the population aged 25 to 64 years, relative increases were highest among the American Indian or Alaska Native (1.45 [95% CI, 1.42-1.48]) Hispanic (1.40 [95% CI, 1.38-1.42]), and Native Hawaiian or Other Pacific Islander (1.39 [95% CI, 1.34-1.44]) groups. There were approximately 13.1 million YPLL among persons aged 25 to 64 years (eTable 2 in Supplement 1). Greater YPLL per capita (and per excess death) among American Indian or Alaska Native, Black, Hispanic, and Native Hawaiian or Other Pacific Islander populations were observed (eTable 4 and 5 in Supplement 1), reflecting younger mean and median ages of decedents in these groups (eFigure 4 and 5 in Supplement 1), compared with Asian and White populations. More than 454 000 (32.9%) excess deaths occurred in people aged 0 to 64 years (Table 1), accounting for approximately 14.2 million (61.2%) of the overall YPLL (Table 2).

#### Ages 65 Years and Older

The older population experienced highest incident rates of excess mortality (eTable 3 in Supplement 1). While the highest excess mortality incidence rates for all groups occurred among persons aged 65 years and older (eTable 1, eTable 2, and eTable 3 in Supplement 1), the observed-to-expected mortality ratios were lower for each race and ethnicity in older adults compared with adults ages 25 to 64 years. Adults ages 65 years accounted for 67% of excess mortality in the overall US population, although in the American Indian or Alaska Native and Native Hawaiian or Other Pacific Islander populations only, people ages younger than 65 years accounted for the majority of the excess mortality (60.5% and 70.3%, respectively) (Table 1).

### COVID-19 and All-Cause Excess Mortality

**Figure 2.**
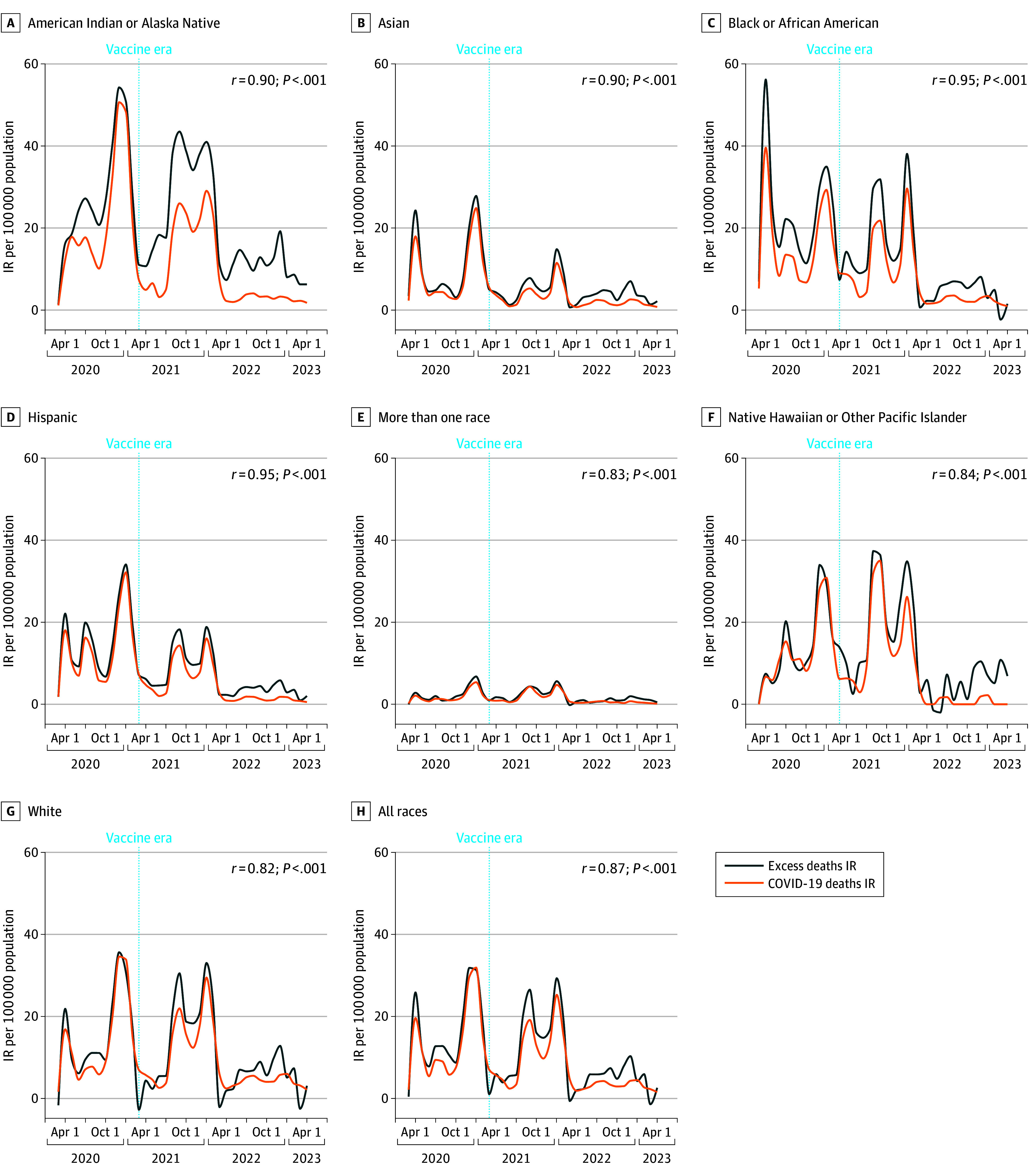
Monthly All-Cause Excess Mortality and COVID-19–Specific Mortality by Race and Ethnicity for All Ages The vertical dashed lines show the start of the vaccine era (using March 2021). Blue lines show excess mortality; orange lines show COVID-19-specific mortality. The Spearman correlation coefficient relating the lines are shown for each race and ethnicity, with their corresponding *P* values. IR indicates incident rate.

During the prevaccine era, the magnitude of excess mortality overall (Figure 2) and within age groups (eFigure 6 in Supplement 1) was greater compared with the vaccine era. Monthly all-cause excess mortality was strongly correlated with COVID-19-specific mortality for the total population (Figure 2) and among adults ages 25 to 64 years and greater than 65 years (eFigures 8 and 9 in Supplement 1). A weak correlation was observed between all-cause excess mortality and COVID-19-specific mortality for the total population aged younger than 25 years (eTable 6 and eFigure 7 in Supplement 1).

COVID-19 mortality was moderately or strongly correlated with death from several medical underlying causes (*ICD-10* chapters) (eFigure 7 in Supplement 1) and varied by race and ethnicity and age (eTable 7 in Supplement 1). Excess mortality for external manner deaths (eg, accidents, homicide, unintentional overdose) was observed for all race and ethnicity groups. No significant correlations were detected between external manner deaths and COVID-19 mortality (eTable 7 in Supplement 1).

### Changes in All-Cause Mortality Disparities During the Pandemic

**Figure 3.**
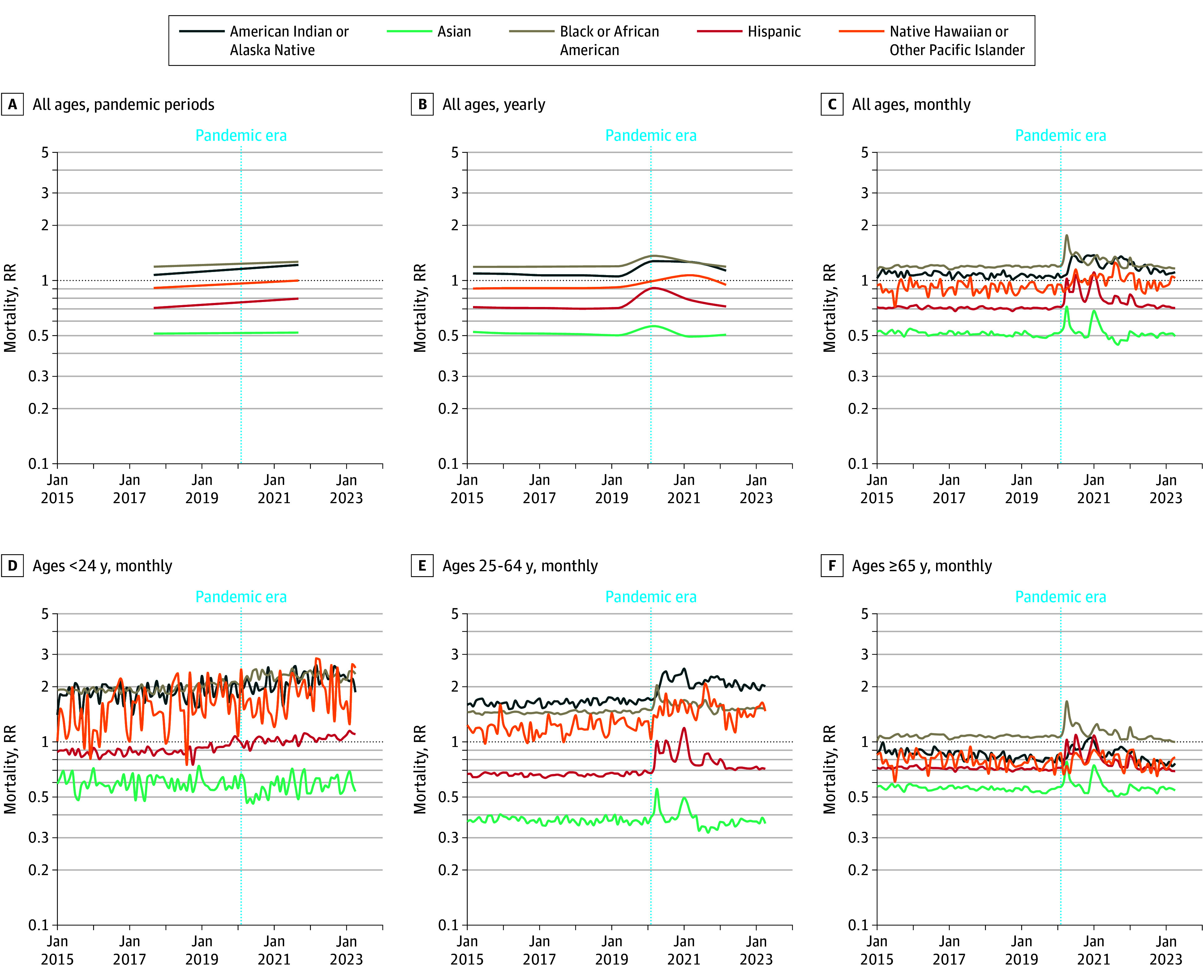
Relative Risk (RR) of Age-Adjusted All-Cause Mortality Compared With Same-Age White Population The dashed vertical lines denote the start of the COVID-19 pandemic period. A, Prepandemic and postpandemic mortality RRs for all ages. B, all ages, by year. C, all ages, by month. Monthly mortality RRs by ages 0-24 years (D), ages 25-64 years (E), and ages 65 years and older (F). American Indian and Alaska Native is shown in blue, Asian in green, Black in gray, Hispanic in red, Native Hawaiian and Other Pacific Islander in orange. 95% CIs are shown for each line compared with the White population. Some CIs may be imperceptible due to narrow bandwidths.

Established largely stable prepandemic disparities in all-cause mortality by race and ethnicity (age-adjusted RRs) acutely changed at the outset of the COVID-19 PHE (Figure 3). Baseline mortality disparities were greatest among older adults, as were pandemic-associated increases (Figure 3; eTable 8 in Supplement 1).

Compared with the White population referent, American Indian or Alaska Native and Black populations experienced the greatest disparities during both the prepandemic and pandemic phases. Among the American Indian or Alaska Native population, the prepandemic RR (1.07 [95% CI, 1.06-1.08]) increased to 1.22 (95% CI, 1.21-1.22) (eTables 8-13 in Supplement 1). Among the Black population, the prepandemic RR (1.19 [95% CI, 1.19-1.19]) increased to 1.26 (95% CI, 1.26-1.27). Among the Asian, Hispanic, and Native Hawaiian or Other Pacific Islander groups (ie, those with favorable prepandemic baselines relative to the White population), all-cause mortality RRs increased during the pandemic. Notable age-specific and temporal observations include the following.

#### Ages Younger Than 25 Years

The Black population had the highest mortality disparity at baseline (1.96 [95% CI, 1.94-1.97]) and the largest RR increase during the pandemic (2.31 [95% CI, 2.29-2.33]) (Figure 3D; eTable 8 in Supplement 1).

#### Ages 25 to 64 Years

The American Indian or Alaska Native population experienced the highest prepandemic mortality disparity (1.64 [95% CI, 1.62-1.66]), and the largest RR increase (2.10 [95% CI, 2.08-2.13]) (Figure 3E; eTable 8 in Supplement 1).

#### Ages 65 Years or Older

Significant increases from baseline RRs occurred in 2 groups: Black (prepandemic: RR, 1.07 [95% CI, 1.07-1.07] vs pandemic: RR, 1.11 [95% CI, 1.11-1.12]) and Hispanic (pre-pandemic RR, 0.72 [95% CI, 0.71-0.72] vs pandemic RR, 0.79 [95% CI, 0.79-0.79]) (Figure 3, Panel F).

#### Temporal Observations

Changes in prepandemic disparities were greater during obvious COVID-19 waves (Figure 3C, 3E, and 3F). Changes in disparities peaked during the first or second pandemic year and then receded (eTable 10 in Supplement 1), but among the population aged younger than 25 years, mortality RR continued to rise (or remained stably elevated) compared with prepandemic baselines (Figure 3D; eTable 11 in Supplement 1). By the third pandemic year, RRs for mortality had returned to prepandemic levels except for American Indian or Alaska Native and Native Hawaiian or Other Pacific Islander populations (Figure 3; eTables 10-13 in Supplement 1). In analyses of the entire population, changes from prepandemic baselines were mostly abolished during the vaccine era (eTable 9 in Supplement 1).

## Discussion

This study documents massive mortality disparities during the US COVID-19 PHE, accounting for 252 000 more excess deaths and 5.2 million more YPLL than would have occurred without these disparities. Though all racial and ethnic groups experienced pandemic-associated excess mortality, American Indian or Alaska Native people experienced the highest rate of all-cause excess mortality and the greatest disparity; their share of excess mortality was nearly twice what would have been projected, based on population shares. Age-stratified analyses revealed important disparities in the younger-than-65 population overall, with the largest increases in mortality (observed-to-expected mortality ratios) occurring among adults ages 25 to 64 years in all race and ethnicity groups. In the population aged younger than 25 years, Black people comprised a majority (51%) of all-cause excess deaths despite representing 14% of the demographic population. Excess mortality among those younger than 25 years was documented among American Indian or Alaska Native, Hispanic, and Native Hawaiian or Pacific Islander but not among the Asian or White populations (Figure 1). Additionally, a long-standing (prepandemic) finding of lower all-cause mortality rate in the Hispanic population compared with White and Asian population was narrowed and even reversed during COVID-19 waves.^17^

This analysis, to our knowledge, extends the existing literature in several ways. First, we used age-stratified component excess mortality modeling, rather than age-adjustment and/or standardization, which identified important differences in excess mortality by race and ethnicity, particularly among the population younger than 65 years; this method allowed us to more accurately characterize conditions experienced by patients, permitted YPLL estimates within racial and ethnic groups, and implied profound, long-term downstream effects related to excess mortality disparities in the younger demographics. Second, these data offer detailed accounts of disparities which spanned the entire pandemic, adding context to previous work.^18,19^ Despite early awareness of mortality disparities, some persisted—albeit disparities decreased especially markedly older populations after vaccines became available (and after the initial Omicron wave) (eFigures 1-3, eTables 8-13 in Supplement 1). Third, we used prepandemic and pandemic age-adjusted, all-cause mortality rates to establish that existing mortality disparities among certain groups widened during the COVID-19 era, rather than merely having been amplified by universal increases in mortality incident rates, an effect especially pronounced among working-aged (ages 25-64) adults. Known COVID-19 waves were associated with sudden changes in disparities.^20^ Fourth, we conducted exploratory analyses that identified contemporaneous increases in non-COVID-19 causes of mortality during the pandemic; increases in some medical causes of death correlated with COVID-19 mortality waves, which implied that many non-COVID-19 deaths were directly or indirectly caused either by the virus or secondary effects. The result, to our knowledge, is the most comprehensive assessment of US COVID-19 pandemic-associated mortality disparities by race and ethnicity and age.

Importantly, we demonstrate that the pandemic appears to have exacerbated historical mortality disparities that have long been understood to reflect strata in social determinants of health, structural inequality, and racism, and which have persisted.^21,22^ Compounding the inherent value of the loss of life/life-years, associated economic productivity losses resulting from identified excess mortality in working-aged and younger adults have important implications, as health and economic affluence are correlated.^23^ Meanwhile, less dramatic disparities among older populations, before and during the pandemic, may reflect “healthy survivor” effects among groups with higher death rates in younger demographics.

Given that race and ethnicity are social constructs, the magnitude of these findings cannot be explained by genetic differences. Nevertheless, biological (and modifiable) mechanisms must be considered. Possible biological^24^ and sociological explanations for our findings include higher rates of pre-existing conditions associated with poor COVID-19 outcomes, higher probability of viral exposure during the pre-vaccine era (reflecting higher shares of essential workers in certain populations, initial lack of equitable access to COVID-19 testing, decreased remote learning or access to school-implemented mitigation measures, and distrust in public messaging regarding infection prevention), decreased access to health care (including *de facto* segregated systems), and racial biases in COVID-19 treatment^25,26,27,28,29,30^—all manifestations of inequities stemming from structural racism and poverty disparities. Initial skepticism of COVID-19 vaccines related to historical medical mistrust was likely another factor; during the early vaccine rollout, receipt of vaccinations was lower among Black people^31^; however, decreased disparities in all-cause mortality in Black people coincided with increased vaccine uptake later in 2021, suggesting successful messaging from trusted leaders.^32^

To prepare for future pandemics, efforts to protect high-risk groups—utilizing evidence-based policy, equitable distribution of resources, and improving infrastructure—are essential. To achieve this, systemic factors must be addressed. In addition to preparation, just-in-time responses should be directed toward high-risk communities during emergencies (pandemics, natural, or human-caused disasters).^33^ For example, policies providing appropriate and cost-free isolation for essential workers would have prevented chains of COVID-19 transmission to higher-risk people who resided in multi-generation households, especially during pre-vaccine and pre-therapeutic eras.^34,35,36^ Similarly, efforts to increase vaccination uptake and implement effective therapeutics among at-risk populations would have reduced disparities in 2020-2021.^31,37,38,39,40,41,42,43^ The relative success of mass vaccination efforts in 2021 mitigated access barriers but were not replicated during subsequent booster campaigns. Differences in vaccination rates and booster receipt in at-risk persons may partly explain why the American Indian or Alaska Native population specifically experienced higher excess mortality later in the pandemic, as this population predominantly lives in underserved areas where access barriers are more common.^29,30^ Lastly, the pandemic was associated with increases in causes of deaths by external manner (eg, homicide, unintentional overdose, but, notably, not suicide), especially in younger age groups, with marked between-group differences.^44,45^ Unlike several medical causes of death, external manner deaths were not temporally correlated with COVID-19- mortality (eTable 7 in Supplement 1).^46^

### Limitations

This study had limitations. First, calculating all-cause excess mortality relies on estimating expected deaths. Although prepandemic trends would likely have continued without the pandemic, certainty is not possible. Second, race and ethnicity designations relied on CDC WONDER data reflecting observed race and ethnicity, which has known limitations related to observer subjectivity and fluidity.^47,48^ In this case, adjudications may have led to underestimations of some of the disparities described.^49,50^ Third, the exploratory underlying cause and/or manner of death analyses are subject to death certificate inaccuracies. Fourth, instead of more granularity, we defined 3 large age groups to capture generational differences, which may have had small effects on the final estimates. Fifth, some of the mortality differences between racial and ethnic groups we report may be due to differences in the urban/rural distributions, which vary by race and ethnicity, and for which we did not measure or make adjustments.

## Conclusions

In this study of excess mortality by race and ethnicity and age in the US during the COVID-19 PHE, substantial mortality disparities were documented, with the highest relative increases occurring among adults ages 25 to 64 years. Some prepandemic mortality disparities were augmented by the pandemic. While pandemics are inevitable, disparities are not. The need to address the conditions that create health disparities—before the next public health crisis—is evident.

## Notes

Supplement 1. **eTable 1.** Expected, Observed and Excess Deaths by Race/Ethnicity, Ages <25 Years **eTable 2.** Expected, Observed and Excess Deaths by Race/Ethnicity, Ages 25-64 Years **eTable 3.** Expected, Observed and Excess Deaths by Race/Ethnicity, Ages ≥65 Years **eFigure 1.** Cumulative Excess Mortality Per 100,000 Persons by Race/Ethnicity and Age Group **eFigure 2.** Cumulative Excess Mortality (Raw) by Race/Ethnicity and Age Group **eFigure 3.** Monthly Excess Mortality Per 100,000 Persons by Race/Ethnicity and Age Group **eTable 4.** Disparity Rate Ratio Between Share of Excess Deaths and Share of Population, by Race/Ethnicity and Age Group **eTable 5.** Years of Potential Life Lost by Race/Ethnicity and Age Group **eFigure 4.** Share of Years of Potential Life Lost by Race/Ethnicity and Age Group **eFigure 5.** Years of Potential Life Lost by Race/Ethnicity and Age Group **eFigure 6.** Excess Mortality Per 100,000 Persons by Race/Ethnicity and Vaccine Period, Ages 25-64 Years and ≥65 Years **eFigure 7.** Covid-19-Specific and All-Cause Excess Mortality Per 100,000 Persons by Race/Ethnicity, Ages <25 Years, With Spearman Correlations (When Possible) **eFigure 8.** Covid-19-Specific and All-Cause Excess Mortality per 100,000 Persons by Race/Ethnicity, Ages 25-64 Years, With Spearman Correlations **eFigure 9.** Covid-19-Specific and All-Cause Excess Mortality Per 100,000 Persons by Race/Ethnicity, Ages ≥65 Years, With Spearman Correlations **eTable 6.** Pearson Correlation Between All-Cause Excess Mortality and Covid-19-Specific Mortality by Race/Ethnicity and Age Group **eTable 7.** Correlation Between Underlying Cause of Death (ICD-10 Chapter) and COVID-19 Deaths, by Race and Age Group **eTable 8.** Relative Risks (All-Cause Mortality) by Age and Race/Ethnicity, Pre-Pandemic and Pandemic Periods **eTable 9.** Relative risks (All-Cause Mortality) by Age and Race/Ethnicity by Vaccine Period **eTable 10.** Relative Risks by Age and Race/Ethnicity, by Pandemic Year, All Ages **eTable 11.** Relative Risks by Age and Race/Ethnicity, by Pandemic Year, Ages <25 Years **eTable 12.** Relative Risks by Age and Race/Ethnicity, by Pandemic Year, Ages 25-64 Years **eTable 13.** Relative Risks by Age and Race/Ethnicity, by Pandemic Year, Ages ≥65 Years

Supplement 2.
Data Sharing Statement
