# Supplementary material for: Racial and Ethnic Disparities in Age-Specific All-Cause Mortality During the COVID-19 Pandemic
Source: JAMA Netw Open. 2024 Oct 11;7(10):e2438918. doi: 10.1001/jamanetworkopen.2024.38918 (PMC11581672; doi:10.1001/jamanetworkopen.2024.38918)
Supplement: Supplement 1. — eTable 1. Expected, Observed and Excess Deaths by Race/Ethnicity, Ages <25 Years eTable 2. Expected, Observed and Excess Deaths by Race/Ethnicity, Ages 25-64 Years eTable 3. Expected, Observed and Excess Deaths by Race/Ethnicity, Ages ≥65 Years eFigure 1. Cumulative Excess Mortality Per 100,000 Persons by Race/Ethnicity and Age Group eFigure 2. Cumulative Excess Mortality (Raw) by Race/Ethnicity and Age Group eFigure 3. Monthly Excess Mortality Per 100,000 Persons by Race/Ethnicity and Age Group eTable 4. Disparity Rate Ratio Between Share of Excess Deaths and Share of Population, by Race/Ethnicity and Age Group eTable 5. Years of Potential Life Lost by Race/Ethnicity and Age Group eFigure 4. Share of Years of Potential Life Lost by Race/Ethnicity and Age Group eFigure 5. Years of Potential Life Lost by Race/Ethnicity and Age Group eFigure 6. Excess Mortality Per 100,000 Persons by Race/Ethnicity and Vaccine Period, Ages 25-64 Years and ≥65 Years eFigure 7. Covid-19-Specific and All-Cause Excess Mortality Per 100,000 Persons by Race/Ethnicity, Ages <25 Years, With Spearman Correlations (When Possible) eFigure 8. Covid-19-Specific and All-Cause Excess Mortality per 100,000 Persons by Race/Ethnicity, Ages 25-64 Years, With Spearman Correlations eFigure 9. Covid-19-Specific and All-Cause Excess Mortality Per 100,000 Persons by Race/Ethnicity, Ages ≥65 Years, With Spearman Correlations eTable 6. Pearson Correlation Between All-Cause Excess Mortality and Covid-19-Specific Mortality by Race/Ethnicity and Age Group eTable 7. Correlation Between Underlying Cause of Death (ICD-10 Chapter) and COVID-19 Deaths, by Race and Age Group eTable 8. Relative Risks (All-Cause Mortality) by Age and Race/Ethnicity, Pre-Pandemic and Pandemic Periods eTable 9. Relative risks (All-Cause Mortality) by Age and Race/Ethnicity by Vaccine Period eTable 10. Relative Risks by Age and Race/Ethnicity, by Pandemic Year, All Ages eTable 11. Relative Risks by Age and Race/Ethnicity, by Pandemic Year, [file jamanetwopen-e2438918-s001.pdf]

# Supplemental Online Content

Faust JS, Renton B, Bongiovanni T, et al. Racial and ethnic disparities in age-specific all-cause mortality during the COVID-19 pandemic. *JAMA Netw Open.* 2024;7(10):e2438918. doi:10.1001/jamanetworkopen.2024.38918

**eTable 1.** Expected, Observed and Excess Deaths by Race/Ethnicity, Ages <25 Years

**eTable 2.** Expected, Observed and Excess Deaths by Race/Ethnicity, Ages 25-64 Years

**eTable 3.** Expected, Observed and Excess Deaths by Race/Ethnicity, Ages ≥65 Years

**eFigure 1.** Cumulative Excess Mortality Per 100,000 Persons by Race/Ethnicity and Age Group

**eFigure 2.** Cumulative Excess Mortality (Raw) by Race/Ethnicity and Age Group

**eFigure 3.** Monthly Excess Mortality Per 100,000 Persons by Race/Ethnicity and Age Group

**eTable 4.** Disparity Rate Ratio Between Share of Excess Deaths and Share of Population, by Race/Ethnicity and Age Group

**eTable 5.** Years of Potential Life Lost by Race/Ethnicity and Age Group

**eFigure 4.** Share of Years of Potential Life Lost by Race/Ethnicity and Age Group

**eFigure 5.** Years of Potential Life Lost by Race/Ethnicity and Age Group

**eFigure 6.** Excess Mortality Per 100,000 Persons by Race/Ethnicity and Vaccine Period, Ages 25-64 Years and ≥65 Years

**eFigure 7.** Covid-19-Specific and All-Cause Excess Mortality Per 100,000 Persons by Race/Ethnicity, Ages <25 Years, With Spearman Correlations (When Possible)

**eFigure 8.** Covid-19-Specific and All-Cause Excess Mortality per 100,000 Persons by Race/Ethnicity, Ages 25-64 Years, With Spearman Correlations

**eFigure 9.** Covid-19-Specific and All-Cause Excess Mortality Per 100,000 Persons by Race/Ethnicity, Ages ≥65 Years, With Spearman Correlations

**eTable 6.** Pearson Correlation Between All-Cause Excess Mortality and Covid-19-Specific Mortality by Race/Ethnicity and Age Group

**eTable 7.** Correlation Between Underlying Cause of Death (ICD-10 Chapter) and COVID-19 Deaths, by Race and Age Group

© 2024 Faust JS et al. *JAMA Network Open.*

**eTable 8.** Relative Risks (All-Cause Mortality) by Age and Race/Ethnicity, Pre-Pandemic and Pandemic Periods

**eTable 9.** Relative risks (All-Cause Mortality) by Age and Race/Ethnicity by Vaccine Period

**eTable 10.** Relative Risks by Age and Race/Ethnicity, by Pandemic Year, All Ages

**eTable 11.** Relative Risks by Age and Race/Ethnicity, by Pandemic Year, Ages <25 Years

**eTable 12.** Relative Risks by Age and Race/Ethnicity, by Pandemic Year, Ages 25-64 Years

**eTable 13.** Relative Risks by Age and Race/Ethnicity, by Pandemic Year, Ages ≥65 Years

This supplemental material has been provided by the authors to give readers additional information about their work.

© 2024 Faust JS et al. *JAMA Network Open.*

## eMethods. Supplemental Methods.

**Excess Mortality**

Population estimates: Projected yearly changes were divided by 12 and applied to each calendar month to achieve smooth changes at the start of each year. The smoothed projected monthly populations for each group were applied to the mortality sARIMA model as a covariate to overcome stationarity because the number of expected deaths is dynamically influenced by the number of people in each group at any time. Monthly projections enabled the capture of seasonal trends.

We computed monthly and cumulative excess mortality incidence rates (per 100,000, using mean pandemic population for each group), and observed-to-expected mortality ratios were determined for all available age groups and race/ethnicity groups. A growing/rolling study period was created for each successive month during the 38-month study period for cumulative excess mortality. For example, March of 2020 was a study period, March-April of 2020 was a study period, meaning that the 38th study period (March 2020-April 2023) encompassed the entire study period. In addition, a comparison of excess mortality was made between the pre-vaccine period and the vaccine period (the vaccine period was measured from March 2021 on for persons ages ≥65 years, and May 2021 for persons ages 25-64).

The monthly 95% confidence interval [CI] boundaries were derived directly from the sARIMA model using the auto.arima function in the R statistical software. The cumulative period CI boundaries were obtained through the 5,000 simulation samples from the estimated sARIMA model for each age group (i.e., for each of the 38 study periods, 95% CIs were separately bootstrapped for each age group within each race/ethnicity and the total all-age composites.

Further, due to the effect of the pandemic on the size of the population, we adjusted the monthly populations to account for cumulative excess mortality during the public health emergency. For 12 months within all years, we defined the projected population for three age groups within each race/ethnicity group and age group (ages <25, 25-64 and ≥65 years), (denoted by $k$, where $1 \le k \le 4$) in month $i$ of 2020 was denoted as $n_{i,k}$; the original point estimate for all-cause deaths was denoted as $\widehat{m}_{i,k}$; the sum of all previous months' excess death were denoted as $P_{i-1,k}$ (starting from March 2020). Therefore, the corrected expected deaths for month $i$ (from March 2020) after correction ($\widehat{m}_{i,k\_adj}$) satisfies:

$$\widehat{m}_{i,k\_adj} = \left(n_{i,k} - P_{i-1,k}\right) \times \frac{\widehat{m}_{i,k}}{n_{i,k}}$$

The above equation also holds for the study period months in 2021-2023, where $P_{i-1,k}$ denotes the sum of all previous months' excess death.

**Race and ethnicity determination**

Race and ethnicity determination relied on CDC WONDER reporting. Per the CDC National Center for Health Statistics (NCHS), race/ethnicity determinations in WONDER typically reflect inventory entered by funeral directors, typically with input from the next-of-kin. Standards and limitations (including issues surrounding underreporting are described by the CDC/NCHS.[1,2]

In addition, due to space considerations, we refer to the racial and ethnic groups without qualification, though the most correct descriptions would be "among people (or decedents) identified as…," reflecting the described subjectivity inherent to the database utilized.

**Model validation**

To test accuracy, sARIMA models were trained on monthly mortality data and population estimates as described (2012-2016 baseline) to project expected deaths from March 2017-February 2020. Because single-race data are not available for these years, the module was tested on alternate data (a 72-component model of the US assembled from 9 Census Divisions, 4 age groups, and 2 genders). In the 3-year validation period, the observed to expected ratio was >0.99 (8,518,962 modeled expected deaths, 8,575,330 observed deaths); 50% of monthly expected values were within ±3% of the unblinded observed values, 89% of monthly expected values were within ±5%, and 100% of monthly expected values were within ±10%. Yearly and total performance are shown below.

**Methods Table**

**Model validation**

| | Year 1 | Year 2 | Year 3 | Total |
|---|---|---|---|---|
| Within 3% | 67% | 33% | 50% | 50% |
| Within 5% | 83% | 92% | 92% | 89% |
| Within 10% | 100% | 100% | 100% | 100% |
| Observed Expected Ratio | 1.01 | 0.98 | 0.99 | 0.99 |
| No. modeled expected deaths | 2,803,351 | 2,863,787 | 2,908,192 | 8,575,330 |
| No. observed deaths | 2,840,464 | 2,805,787 | 2,872,711 | 8,518,962 |

**Potential Excess Deaths Averted.**

The number of deaths averted was calculated by subtracting the observed-to-expected all-cause mortality ratio in the same-age White population from the observed-to-expected ratio for each individual population and multiplying the difference by modeled expected deaths that group (a second comparison using the same-age Asian population was also done).[3]

**Years of Potential Life Lost (YPLL) and Potential YPLL Averted.**

We assumed that within a 10-year age group, the distributions of excess deaths for each single year were like those seen among observed deaths during the pandemic period. Because no tables are available for NHPI or More than one race groups, we provided a range using the greatest (Asian) and least (AI/AN) life table values found among the other groups. For excess deaths averted and YPLL averted, the life expectancy estimates from Asian population were used.

© 2024 Faust JS et al. *JAMA Network Open.*

Further, to determine the average YPLL per excess death, we divided the calculated YPLL by modeled excess deaths for each demographic.

The share of excess deaths and years of potential life lost from each of the three large age groups was determined for each race/ethnicity and age group by dividing the number of excess deaths in each age group and dividing by the total excess deaths for that race/ethnicity. For the share calculations (but not elsewhere), negative excess mortality was considered as 0.

Potential YPLL averted was calculated by applying the number of excess deaths averted (as above) to life expectancy tables (as above).

**Disparity Rate Ratios**

To quantify whether mortality shares exceeded population shares in any racial or ethnic groups, we calculated "disparity rate ratios" (DRR) during the pandemic by dividing the share of the excess mortality and YPLL of each race/ethnicity, within each age group, by the population share within that age group. 95% CIs were determined using bandwidths from the excess mortality model. For example, for the all-ages analysis, AI/AN people represented 1.4% of excess mortality, which comprising slightly under 0.7% of the overall population. Therefore, the DRR=1.4%/0.7=1.96 with 95% confidence intervals (1.82-2.11) reflecting the corresponding excess mortality bandwidth.

**Cause-Specific Excess Mortality**

The cause-specific excess mortality module was generated using a similar approach as the all-cause excess mortality model, with two exceptions due to data limitations. First, we did not correct for the lower-than-expected population during the pandemic, owing to cumulative excess mortality; Second, the pre-pandemic baseline period was only available for 2018-2020, due to changes in the CDC's method of reporting mortality data (i.e., the transition from bridged to single races/ethnicity).

We modeled cause-specific expected monthly deaths for each race/ethnicity and age group during the study period according to the International Classification of Diseases, Tenth Revision (ICD-10), limiting our analysis for each race/ethnicity and age group to causes for which the average monthly deaths were >25 during the pre-pandemic period, to avoid modeling causes of death with suppressed data (CDC does not report monthly causes of death whose counts are 1-9 deaths). A heat map was produced from the excess death incidence rate for each month. Total excess mortality from each modeled cause was determined (per 100,000 people).

© 2024 Faust JS et al. *JAMA Network Open.*

ICD-10 chapters in CDC WONDER[4] are as follows:

| Chapter Title | ICD-10 Codes |
|---|---|
| Certain Infectious and Parasitic Diseases | A00-B99 |
| Neoplasms | C00-D48 |
| Diseases of the Blood and Blood-Forming Organs and Certain Disorders Involving the Immune M | D50-D89 |
| Endocrine, Nutritional and Metabolic Diseases | E00-E89 |
| Mental and Behavioral Disorders | F01-F99 |
| Diseases of the Nervous System | G00-G99 |
| Diseases of the Eye and Adnexa | H00-H59 |
| Diseases of the Ear and Mastoid Process | H60-H93 |
| Diseases of the Circulatory System | I00-I99 |
| Diseases of the Respiratory System | J00-J99 |
| Diseases of the Digestive System | K00-K92 |
| Diseases of the Skin and Subcutaneous Tissue | L00-L98 |
| Diseases of the Musculoskeletal System and Connective Tissue | M00-M99 |
| Diseases of the Genitourinary System | N00-N98 |
| Pregnancy, Childbirth and the Puerperium | O00-O99 |
| Certain Conditions Originating in the Perinatal Period | P00-P96 |
| Congenital Malformations, Deformations and Chromosomal Abnormalities | Q00-Q99 |
| Symptoms, Signs and Abnormal Clinical and Laboratory Findings, Not Elsewhere Classified | R00-R99 |
| Codes for Special Purposes | U00-U99 |
| External Causes of Morbidity and Mortality | V01-Y98 |

**Spearman correlation strength ranges.**

Spearman correlation strength ranges for measured temporal relationships between all-cause excess mortality and cause-specific mortality were as follows[5]:

| | |
|---|---|
| 0-0.19 | very weak |
| 0.2-0.39 | weak |
| 0.4-0.59 | moderate |
| 0.6-0.79 | strong |
| 0.8-1 | very strong |

References:


1. Arias E, Heron M, Ni H. The Validity of Race and Hispanic-origin Reporting on Death Certificates in the United States: An Update. *Vital Health Stat*. 2(2016 Aug 1;(172):1-21.):29.

2. CDC/NCHS. Personal Communication.

3. Renton B, Du C, Chen AJ, et al. State-Level Excess Mortality and Potential Deaths Averted in US Adults During the Delta and Omicron Waves of COVID-19. *J Gen Intern Med*. 2024;39(1):142-146. doi:10.1007/s11606-023-08374-2


© 2024 Faust JS et al. *JAMA Network Open.*

4. National Center for Health Statistics. CDC WONDER. Accessed August 25, 2023. https://wonder.cdc.gov/

5. 11. Correlation and regression | The BMJ. The BMJ | The BMJ: leading general medical journal. Research. Education. Comment. October 28, 2020. Accessed August 11, 2024. https://www.bmj.com/about-bmj/resources-readers/publications/statistics-square-one/11-correlation-and-regression

© 2024 Faust JS et al. *JAMA Network Open.*

## Supplemental Exhibits:

**eTable 1.** Expected, observed and excess deaths by race/ethnicity, ages <25 years.

### Expected and Observed Deaths by Race

| Race | Age Group | Expected deaths (95% CI) | Observed Deaths | Excess deaths, no (95% CI) | Excess deaths, IR (95% CI) | Ratio of observed to expected deaths (95% CI) | Potential Deaths Averted - White as Reference | YPLL | Potential YPLL Averted | Population/YPLL | YPLL/Excess |
|---|---|---|---|---|---|---|---|---|---|---|---|
| American Indian or Alaska Native | <25 years | 2,721 (2,610 - 2,832) | 3,164 | 443 (332 - 554) | 53.14 (39.84 - 66.45) | 1.16 (1.12 - 1.21) | 378 (85%) | 23,774 | 20,265 (85%) | 35.1 | 53.7 |
| Asian | <25 years | 5,426 (4,868 - 5,984) | 5,499 | 73 (-485 - 631) | 1.34 (-8.85 - 11.53) | 1.01 (0.92 - 1.13) | -57 (-78%) | 5,333 | -4,146 (-78%) | 1,026.9 | 72.7 |
| Black or African American | <25 years | 47,896 (45,657 - 50,136) | 56,890 | 8,994 (6,754 - 11,233) | 63.89 (47.98 - 79.81) | 1.19 (1.13 - 1.25) | 7,842 (87%) | 542,891 | 473,398 (87%) | 25.9 | 60.4 |
| Hispanic | <25 years | 40,758 (38,478 - 43,038) | 46,734 | 5,976 (3,696 - 8,256) | 23.17 (14.33 - 32.01) | 1.15 (1.09 - 1.21) | 4,996 (84%) | 395,015 | 330,256 (84%) | 65.3 | 66.1 |
| More than one race | <25 years | 5,982 (5,836 - 6,129) | 5,894 | -88 (-235 - 58) | -1.98 (-5.26 - 1.29) | 0.99 (0.96 - 1.01) | -232 (263%) | -6,557 | -17,215 (263%) | -680.2 | 74.1 |
| Native Hawaiian or Other Pacific Islander | <25 years | 576 (529 - 623) | 680 | 104 (57 - 151) | 49.13 (26.9 - 71.36) | 1.18 (1.09 - 1.28) | 90 (87%) | 7,548 | 6,541 (87%) | 28 | 72.8 |
| White | <25 years | 87,590 (77,740 - 97,440) | 89,695 | 2,105 (-7,745 - 11,955) | 4.11 (-15.1 - 23.31) | 1.02 (0.92 - 1.15) | Reference | 136,959 | Reference | 374.4 | 65.1 |
| All races | <25 years | 190,950 (152,052 - 229,847) | 208,556 | 17,606 (-21,291 - 56,504) | 17.24 (-20.85 - 55.33) | 1.09 (0.91 - 1.37) | 13,017 (74%) | 1,104,723 | 848,318 (77%) | 92.4 | 62.7 |

**eTable 2.** Expected, observed and excess deaths by race/ethnicity, ages 25-64 years.

### Expected and Observed Deaths by Race

| Race | Age Group | Expected deaths (95% CI) | Observed Deaths | Excess deaths, no (95% CI) | Excess deaths, IR (95% CI) | Ratio of observed to expected deaths (95% CI) | Potential Deaths Averted - White as Reference | YPLL | Potential YPLL Averted | Population/YPLL | YPLL/Excess |
|---|---|---|---|---|---|---|---|---|---|---|---|
| American Indian or Alaska Native | 25-64 years | 25,732 (25,199 - 26,265) | 37,394 | 11,662 (11,129 - 12,195) | 919.5 (877.46 - 961.54) | 1.45 (1.42 - 1.48) | 7,814 (67%) | 308,062 | 205,364 (67%) | 4.1 | 26.4 |
| Asian | 25-64 years | 49,022 (48,334 - 49,711) | 60,529 | 11,507 (10,818 - 12,195) | 102.28 (96.16 - 108.4) | 1.23 (1.22 - 1.25) | 4,177 (36%) | 356,746 | 139,792 (39%) | 31.7 | 34.2 |
| Black or African American | 25-64 years | 408,133 (398,137 - 418,129) | 505,432 | 97,299 (87,303 - 107,295) | 439.38 (394.24 - 484.52) | 1.24 (1.21 - 1.27) | 36,273 (37%) | 2,534,603 | 957,469 (38%) | 8.8 | 27 |
| Hispanic | 25-64 years | 242,622 (238,744 - 246,499) | 340,066 | 97,444 (93,567 - 101,322) | 307.85 (295.6 - 320.1) | 1.40 (1.38 - 1.42) | 61,167 (63%) | 2,993,509 | 1,923,511 (64%) | 10.6 | 31.4 |
| More than one race | 25-64 years | 17,303 (16,988 - 17,618) | 20,762 | 3,459 (3,144 - 3,774) | 120.95 (109.95 - 131.96) | 1.20 (1.18 - 1.22) | 872 (25%) | 160,808 | 32,505 (20%) | 17.8 | 37.9 |
| Native Hawaiian or Other Pacific Islander | 25-64 years | 5,037 (4,868 - 5,205) | 6,987 | 1,950 (1,782 - 2,119) | 570.63 (521.32 - 619.94) | 1.39 (1.34 - 1.44) | 1,197 (61%) | 73,263 | 42,063 (57%) | 4.6 | 36.2 |
| White | 25-64 years | 1,426,458 (1,335,794 - 1,517,121) | 1,639,747 | 213,289 (122,626 - 303,953) | 210.19 (120.84 - 299.53) | 1.15 (1.08 - 1.23) | Reference | 6,743,271 | Reference | 15.1 | 29 |
| All races | 25-64 years | 2,174,307 (2,133,020 - 2,215,594) | 2,610,917 | 436,610 (395,323 - 477,897) | 255.34 (231.19 - 279.48) | 1.20 (1.18 - 1.22) | 111,500 (26%) | 13,104,482 | 3,222,866 (24%) | 13.1 | 29.1 |

© 2024 Faust JS et al. *JAMA Network Open.*

**eTable 3.** Expected, observed and excess deaths by race/ethnicity, ages ≥65 years.

**Expected and Observed Deaths by Race**

| Race | Age Group | Expected deaths (95% CI) | Observed Deaths | Excess deaths, no (95% CI) | Excess deaths, IR (95% CI) | Ratio of observed to expected deaths (95% CI) | Potential Deaths Averted - White as Reference | YPLL | Potential YPLL Averted | Population/YPLL | YPLL/Excess |
|---|---|---|---|---|---|---|---|---|---|---|---|
| American Indian or Alaska Native | 65+ years | 30,750 (29,839 - 31,660) | 38,656 | 7,906 (6,996 - 8,817) | 2,383.83 (2,109.27 - 2,658.4) | 1.26 (1.22 - 1.30) | 4,337 (55%) | 73,880 | 43,167 (58%) | 4.5 | 10 |
| Asian | 65+ years | 186,943 (182,412 - 191,475) | 223,344 | 36,401 (31,869 - 40,932) | 1,316.21 (1,152.36 - 1,480.05) | 1.19 (1.17 - 1.22) | 14,704 (40%) | 396,683 | 146,099 (37%) | 7 | 10.7 |
| Black or African American | 65+ years | 693,040 (677,061 - 709,020) | 815,064 | 122,024 (106,044 - 138,003) | 2,277.07 (1,978.88 - 2,575.26) | 1.18 (1.15 - 1.20) | 41,587 (34%) | 1,211,731 | 385,720 (32%) | 4.4 | 9.5 |
| Hispanic | 65+ years | 439,713 (428,149 - 451,277) | 558,400 | 118,687 (107,123 - 130,251) | 2,325.92 (2,099.3 - 2,552.55) | 1.27 (1.24 - 1.30) | 67,652 (57%) | 1,273,166 | 645,872 (51%) | 4 | 10.3 |
| More than one race | 65+ years | 24,081 (23,038 - 25,124) | 26,367 | 2,286 (1,243 - 3,329) | 471.31 (256.28 - 686.33) | 1.09 (1.05 - 1.14) | -509 (-22%) | 32,157 | -6,089 (-19%) | 15.1 | 14.3 |
| Native Hawaiian or Other Pacific Islander | 65+ years | 6,612 (6,374 - 6,849) | 7,480 | 868 (631 - 1,106) | 1,266.68 (919.7 - 1,613.66) | 1.13 (1.09 - 1.17) | 101 (12%) | 15,150 | 1,338 (9%) | 4.5 | 13.1 |
| White | 65+ years | 5,514,607 (5,360,883 - 5,668,331) | 6,154,649 | 640,042 (486,318 - 793,766) | 1,485.71 (1,128.87 - 1,842.54) | 1.12 (1.09 - 1.15) | Reference | 6,019,491 | Reference | 7.2 | 9.1 |
| All races | 65+ years | 6,895,746 (6,713,642 - 7,077,850) | 7,823,960 | 928,214 (746,110 - 1,110,318) | 1,622.97 (1,304.56 - 1,941.37) | 1.13 (1.11 - 1.17) | 127,873 (14%) | 9,011,342 | 1,121,109 (12%) | 6.3 | 9.4 |

**eFigure 1.** Cumulative excess mortality per 100,000 persons by race/ethnicity and age group. 95% confidence intervals are shown for all categories but may not be visible due to narrow bandwidths.

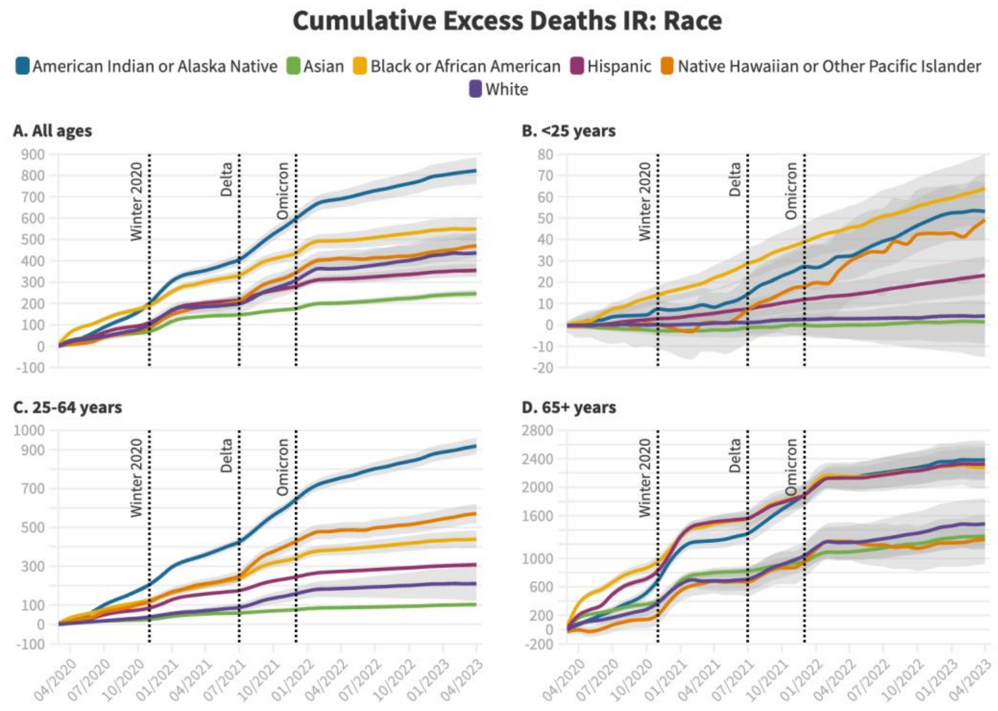


© 2024 Faust JS et al. *JAMA Network Open.*

**eFigure 2.** Cumulative excess mortality (raw) by race/ethnicity and age group. 95% confidence intervals are shown for all categories but may not be visible due to narrow bandwidths.

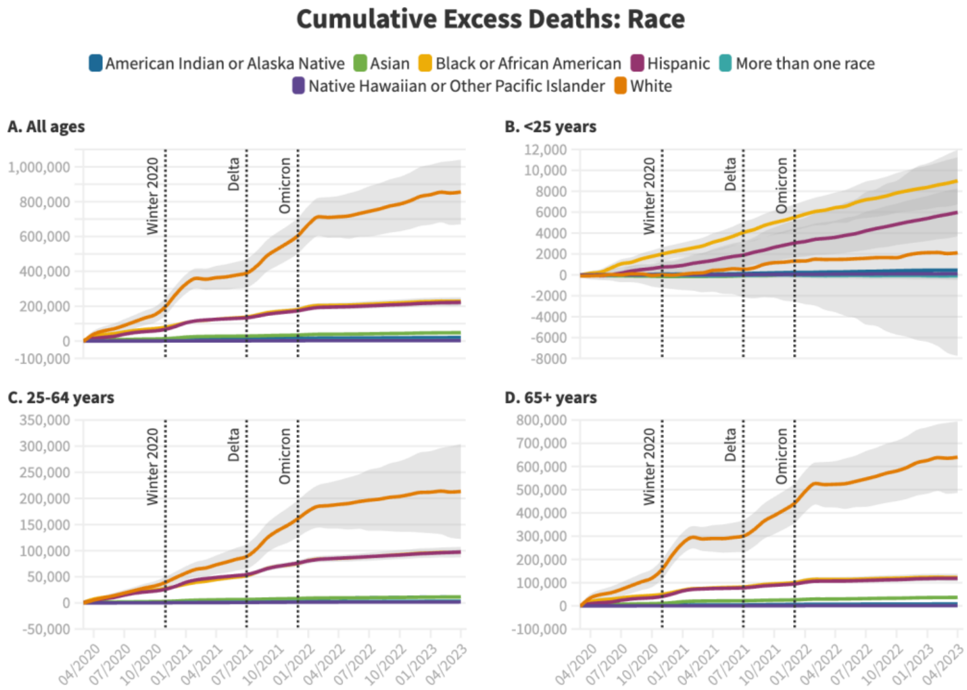


**eFigure 3.** Monthly excess mortality per 100,000 persons by race/ethnicity and age group. 95% confidence intervals are not shown (for clarity).

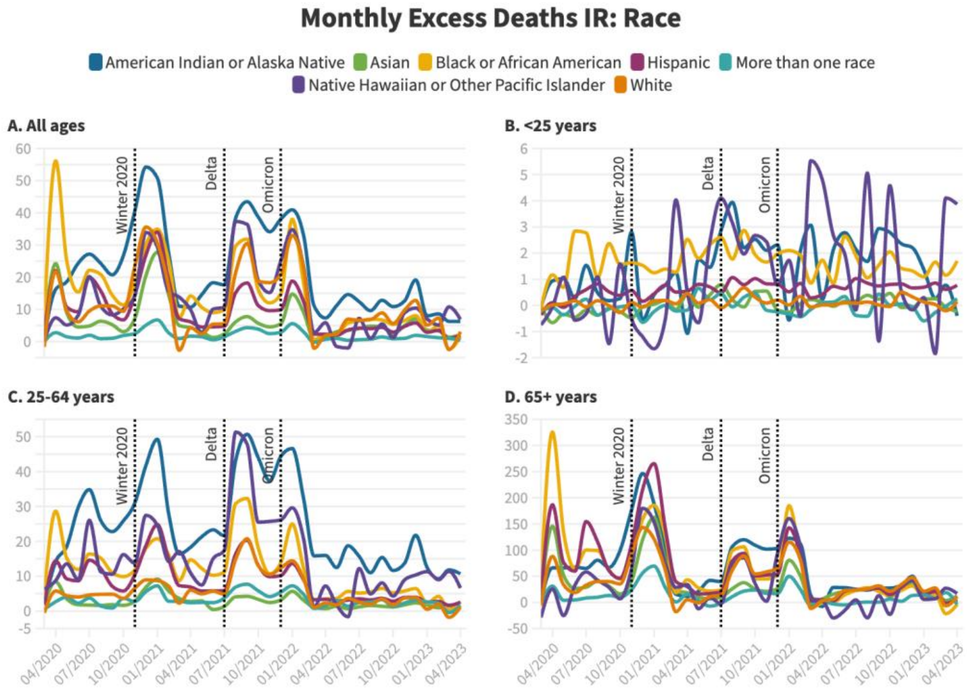


© 2024 Faust JS et al. *JAMA Network Open.*

**eTable 4.** Disparity rate ratio between share of excess deaths and share of population, by race/ethnicity and age group.

**Disparity Rate Ratio**

| Age Group | Race | Percent share of population | Percent share of excess death | Percent share of YPLL | Disparity Rate Ratio | YPLL Disparity Rate Ratio |
|---|---|---|---|---|---|---|
| All ages | American Indian or Alaska Native | 0.7% | 1.4% | 1.7% | 1.96 (1.82 - 2.11) | 2.36 (2.36 - 2.37) |
| All ages | Asian | 5.9% | 3.5% | 3.3% | 0.59 (0.55 - 0.63) | 0.55 (0.55 - 0.55) |
| All ages | Black or African American | 12.6% | 16.5% | 18.4% | 1.31 (1.20 - 1.42) | 1.46 (1.46 - 1.47) |
| All ages | Hispanic | 18.9% | 16.1% | 20% | 0.85 (0.78 - 0.91) | 1.06 (1.05 - 1.06) |
| All ages | More than one race | 2.4% | 0.4% | 0.8% | 0.17 (0.11 - 0.24) | 0.34 (0.33 - 0.34) |
| All ages | Native Hawaiian or Other Pacific Islander | 0.2% | 0.2% | 0.4% | 1.12 (1.00 - 1.25) | 2.19 (2.18 - 2.20) |
| All ages | White | 59.3% | 61.9% | 55.4% | 1.04 (0.94 - 1.12) | 0.93 (0.93 - 0.94) |
| <25 years | American Indian or Alaska Native | 0.8% | 2.5% | 2.2% | 3.08 ( 2.33 - 3.83) | 2.64 ( 2.62 - 2.65) |
| <25 years | Asian | 5.4% | 0.4% | 0.5% | 0.08 (-0.53 - 0.65) | 0.09 ( 0.08 - 0.10) |
| <25 years | Black or African American | 13.8% | 51.1% | 49.1% | 3.71 ( 3.19 - 4.11) | 3.56 ( 3.56 - 3.57) |
| <25 years | Hispanic | 25.3% | 33.9% | 35.7% | 1.34 ( 0.95 - 1.64) | 1.42 ( 1.41 - 1.42) |
| <25 years | More than one race | 4.4% | -0.5% | 0% | -0.12 (-0.31 - 0.07) | 0.00 (-0.14 - -0.13) |
| <25 years | Native Hawaiian or Other Pacific Islander | 0.2% | 0.6% | 0.7% | 2.85 ( 1.56 - 4.13) | 3.30 ( 3.28 - 3.32) |
| <25 years | White | 50.2% | 12% | 12.4% | 0.24 (-1.99 - 0.87) | 0.25 ( 0.23 - 0.26) |
| 25-64 years | American Indian or Alaska Native | 0.7% | 2.7% | 2.3% | 3.60 (3.44 - 3.76) | 3.15 (3.15 - 3.16) |
| 25-64 years | Asian | 6.6% | 2.6% | 2.7% | 0.40 (0.38 - 0.42) | 0.41 (0.41 - 0.41) |
| 25-64 years | Black or African American | 13% | 22.3% | 19.2% | 1.72 (1.58 - 1.86) | 1.49 (1.48 - 1.49) |
| 25-64 years | Hispanic | 18.5% | 22.3% | 22.7% | 1.21 (1.17 - 1.24) | 1.23 (1.23 - 1.23) |
| 25-64 years | More than one race | 1.7% | 0.8% | 1.2% | 0.47 (0.43 - 0.52) | 0.73 (0.73 - 0.73) |
| 25-64 years | Native Hawaiian or Other Pacific Islander | 0.2% | 0.4% | 0.6% | 2.23 (2.04 - 2.43) | 2.78 (2.78 - 2.79) |
| 25-64 years | White | 59.3% | 48.9% | 51.2% | 0.82 (0.60 - 0.97) | 0.86 (0.86 - 0.87) |
| 65+ years | American Indian or Alaska Native | 0.6% | 0.9% | 0.8% | 1.47 (1.30 - 1.64) | 1.41 (1.39 - 1.43) |
| 65+ years | Asian | 4.8% | 3.9% | 4.4% | 0.81 (0.71 - 0.91) | 0.91 (0.90 - 0.92) |
| 65+ years | Black or African American | 9.4% | 13.1% | 13.4% | 1.40 (1.24 - 1.56) | 1.43 (1.42 - 1.45) |
| 65+ years | Hispanic | 8.9% | 12.8% | 14.1% | 1.43 (1.31 - 1.55) | 1.58 (1.57 - 1.59) |
| 65+ years | More than one race | 0.8% | 0.2% | 0.4% | 0.29 (0.16 - 0.42) | 0.42 (0.41 - 0.43) |
| 65+ years | Native Hawaiian or Other Pacific Islander | 0.1% | 0.1% | 0.2% | 0.78 (0.57 - 0.99) | 1.40 (1.38 - 1.42) |
| 65+ years | White | 75.3% | 69% | 66.7% | 0.92 (0.83 - 0.97) | 0.89 (0.88 - 0.89) |

© 2024 Faust JS et al. *JAMA Network Open.*

**eTable 5.** Years of potential life lost (YPLL) by race/ethnicity and age group. For NHPI and More than one race categories, YPLL were determined using life expectancy for the Asian category, because CDC life tables do not include estimates for these populations.

Years of Potential Life Lost by Race and Age Group

| Race | Age Group | Years of Potential Life Lost | Years of Potential Life Lost (All Ages) |
|---|---|---|---|
| American Indian or Alaska Native | <25 years | 23,774 | 405,716 |
| American Indian or Alaska Native | 25-34 years | 67,377 | |
| American Indian or Alaska Native | 35-44 years | 76,559 | |
| American Indian or Alaska Native | 45-54 years | 80,795 | |
| American Indian or Alaska Native | 55-64 years | 83,331 | |
| American Indian or Alaska Native | 65-74 years | 41,812 | |
| American Indian or Alaska Native | 75-84 years | 23,712 | |
| American Indian or Alaska Native | 85+ years | 8,356 | |
| Asian | <25 years | 5,333 | 758,762 |
| Asian | 25-34 years | 51,772 | |
| Asian | 35-44 years | 76,788 | |
| Asian | 45-54 years | 97,705 | |
| Asian | 55-64 years | 130,482 | |
| Asian | 65-74 years | 192,663 | |
| Asian | 75-84 years | 136,734 | |
| Asian | 85+ years | 67,286 | |
| Black or African American | <25 years | 542,891 | 4,289,225 |
| Black or African American | 25-34 years | 451,948 | |
| Black or African American | 35-44 years | 586,194 | |
| Black or African American | 45-54 years | 697,982 | |
| Black or African American | 55-64 years | 798,480 | |
| Black or African American | 65-74 years | 722,152 | |
| Black or African American | 75-84 years | 351,346 | |
| Black or African American | 85+ years | 138,233 | |
| Hispanic or Latino | <25 years | 395,015 | 4,661,690 |
| Hispanic or Latino | 25-34 years | 568,329 | |
| Hispanic or Latino | 35-44 years | 654,832 | |
| Hispanic or Latino | 45-54 years | 871,973 | |
| Hispanic or Latino | 55-64 years | 898,375 | |
| Hispanic or Latino | 65-74 years | 729,481 | |
| Hispanic or Latino | 75-84 years | 396,010 | |
| Hispanic or Latino | 85+ years | 147,675 | |
| More than one race | <25 years | -6,557 | 186,408 |
| More than one race | 25-34 years | 47,109 | |
| More than one race | 35-44 years | 27,854 | |
| More than one race | 45-54 years | 50,699 | |
| More than one race | 55-64 years | 35,146 | |
| More than one race | 65-74 years | 24,961 | |
| More than one race | 75-84 years | 5,044 | |
| More than one race | 85+ years | 2,152 | |
| Native Hawaiian or Other Pacific Islander | <25 years | 7,548 | 95,961 |
| Native Hawaiian or Other Pacific Islander | 25-34 years | 12,226 | |
| Native Hawaiian or Other Pacific Islander | 35-44 years | 20,289 | |
| Native Hawaiian or Other Pacific Islander | 45-54 years | 20,790 | |
| Native Hawaiian or Other Pacific Islander | 55-64 years | 19,957 | |
| Native Hawaiian or Other Pacific Islander | 65-74 years | 8,655 | |
| Native Hawaiian or Other Pacific Islander | 75-84 years | 5,424 | |
| Native Hawaiian or Other Pacific Islander | 85+ years | 1,071 | |
| White | <25 years | 136,959 | 12,899,721 |
| White | 25-34 years | 991,001 | |
| White | 35-44 years | 1,020,781 | |
| White | 45-54 years | 2,138,649 | |
| White | 55-64 years | 2,592,840 | |
| White | 65-74 years | 2,795,299 | |
| White | 75-84 years | 2,377,575 | |
| White | 85+ years | 846,617 | |

**eFigure 4.** Share of years of potential life lost by race/ethnicity and age group (10-year groupings with the exception of <25 and ≥85 years, which were measured as one group to avoid suppression).

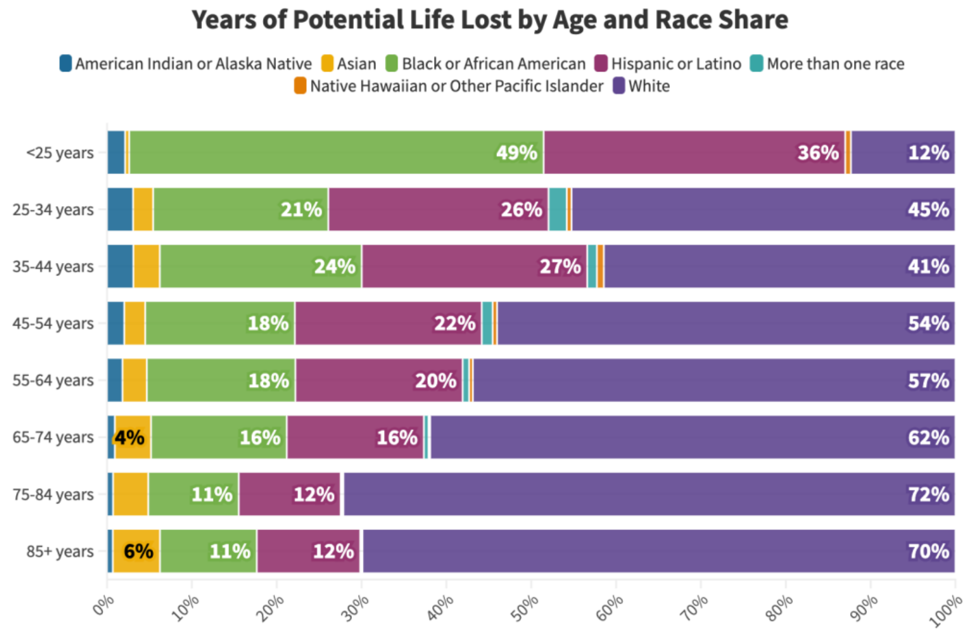


© 2024 Faust JS et al. *JAMA Network Open.*

**eFigure 5.** Years of potential life lost by race/ethnicity and age group. For NHPI and More than one race categories, YPLL were determined using life expectancy for the AI/AN category, because CDC life tables do not include estimates for these populations.

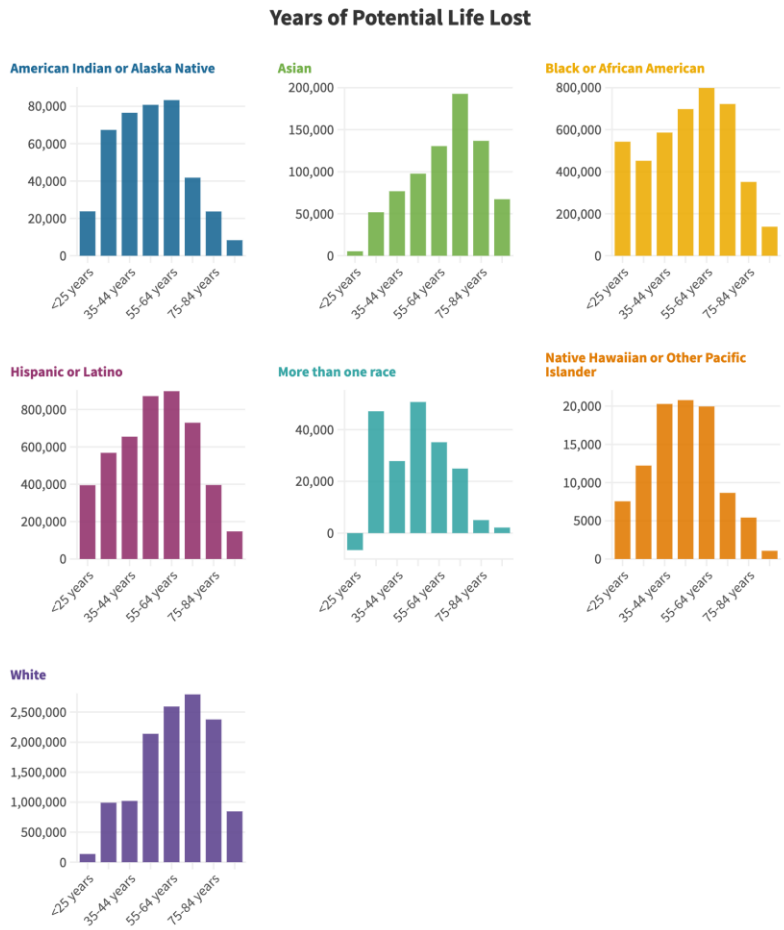

**eFigure 6.** Excess mortality per 100,000 persons by race/ethnicity and vaccine period, ages 25-64 years (left panel) and ≥65 years (right panel). 95% confidence intervals are shown in the yellow bars.

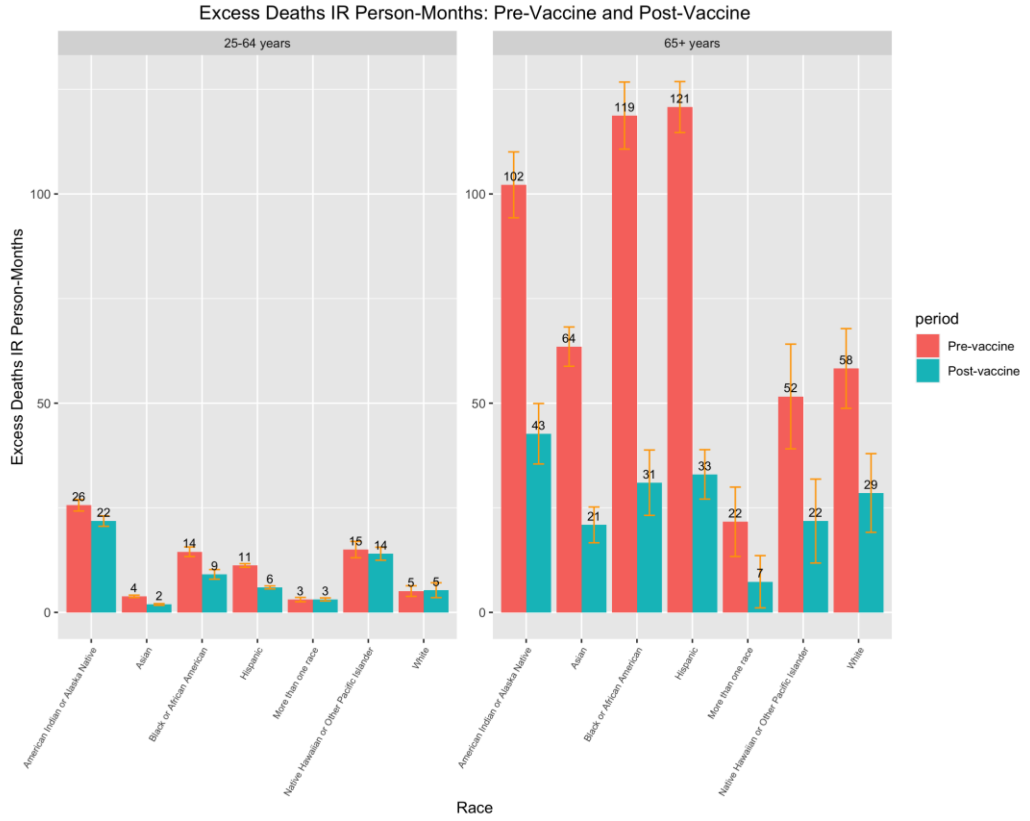


© 2024 Faust JS et al. *JAMA Network Open.*

**eFigure 7.** Covid-19-specific and all-cause excess mortality per 100,000 persons by race/ethnicity, ages <25 years, with Spearman correlations (when possible). Covid-19-specific deaths are not shown in some panels due to suppression or 0 values.

© 2024 Faust JS et al. *JAMA Network Open.*

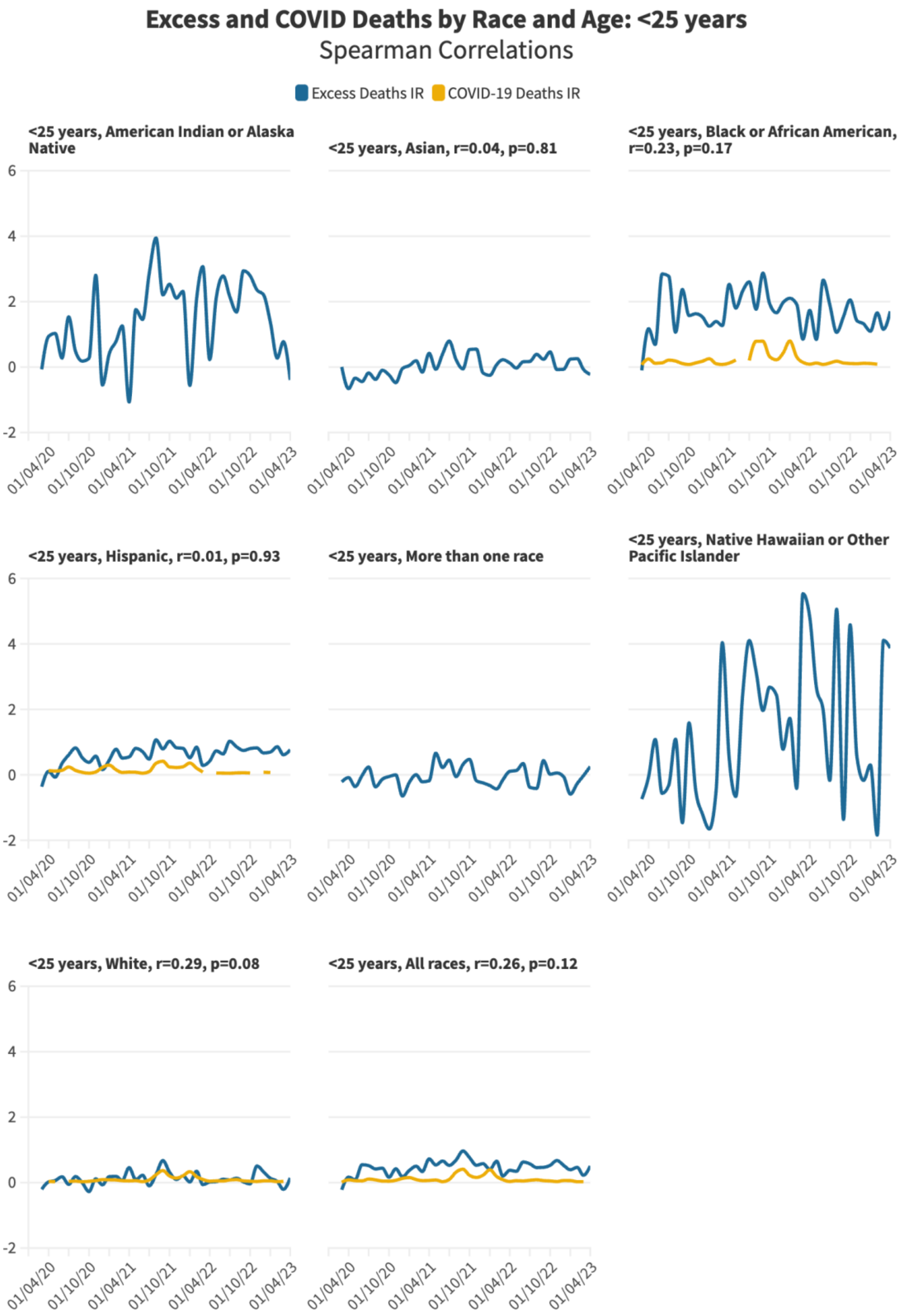


© 2024 Faust JS et al. *JAMA Network Open.*

**eFigure 8.** Covid-19-specific and all-cause excess mortality per 100,000 persons by race/ethnicity, ages 25-64 years, with Spearman correlations.

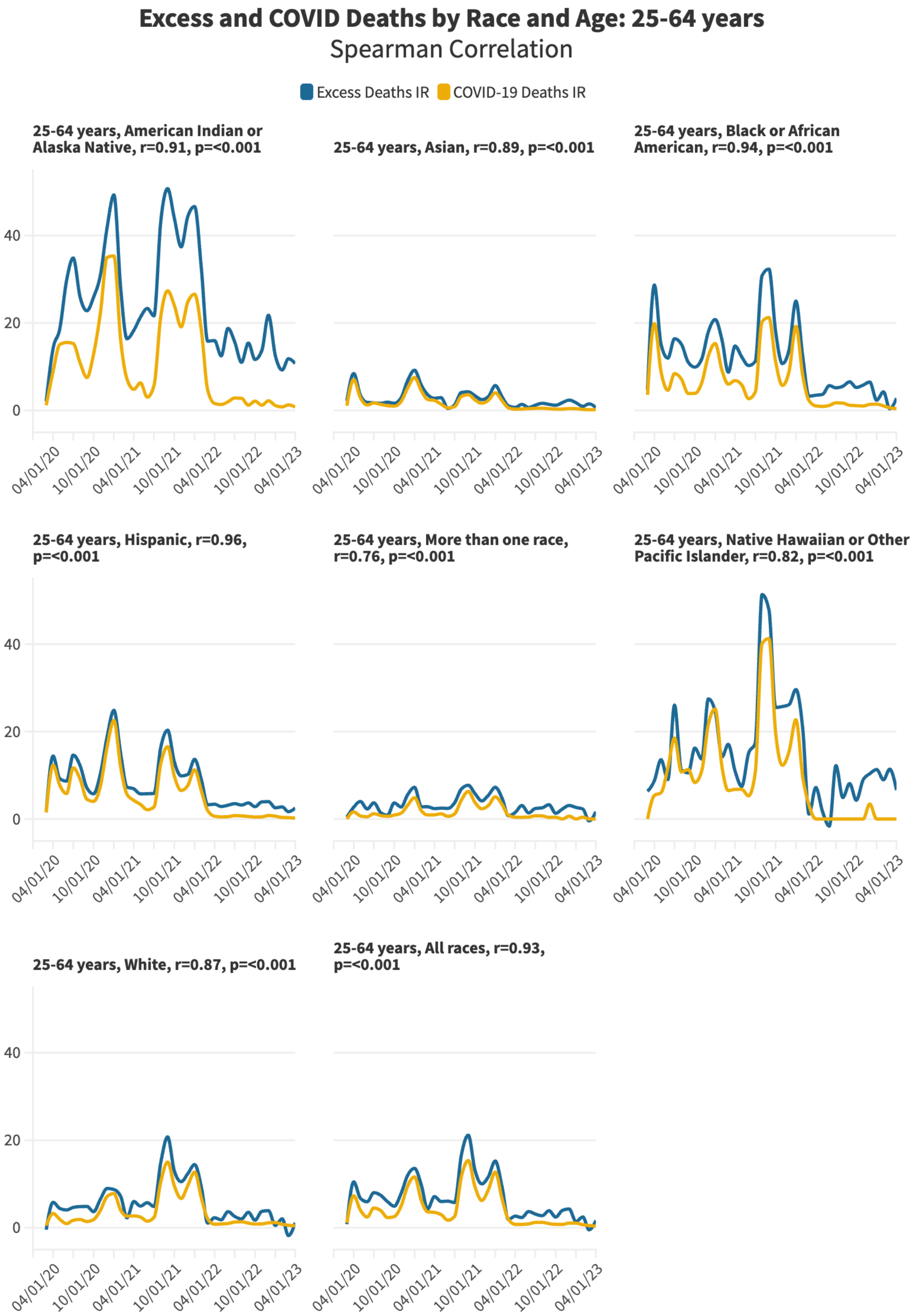


© 2024 Faust JS et al. *JAMA Network Open.*

**eFigure 9.** Covid-19-specific and all-cause excess mortality per 100,000 persons by race/ethnicity, ages ≥65 years, with Spearman correlations.

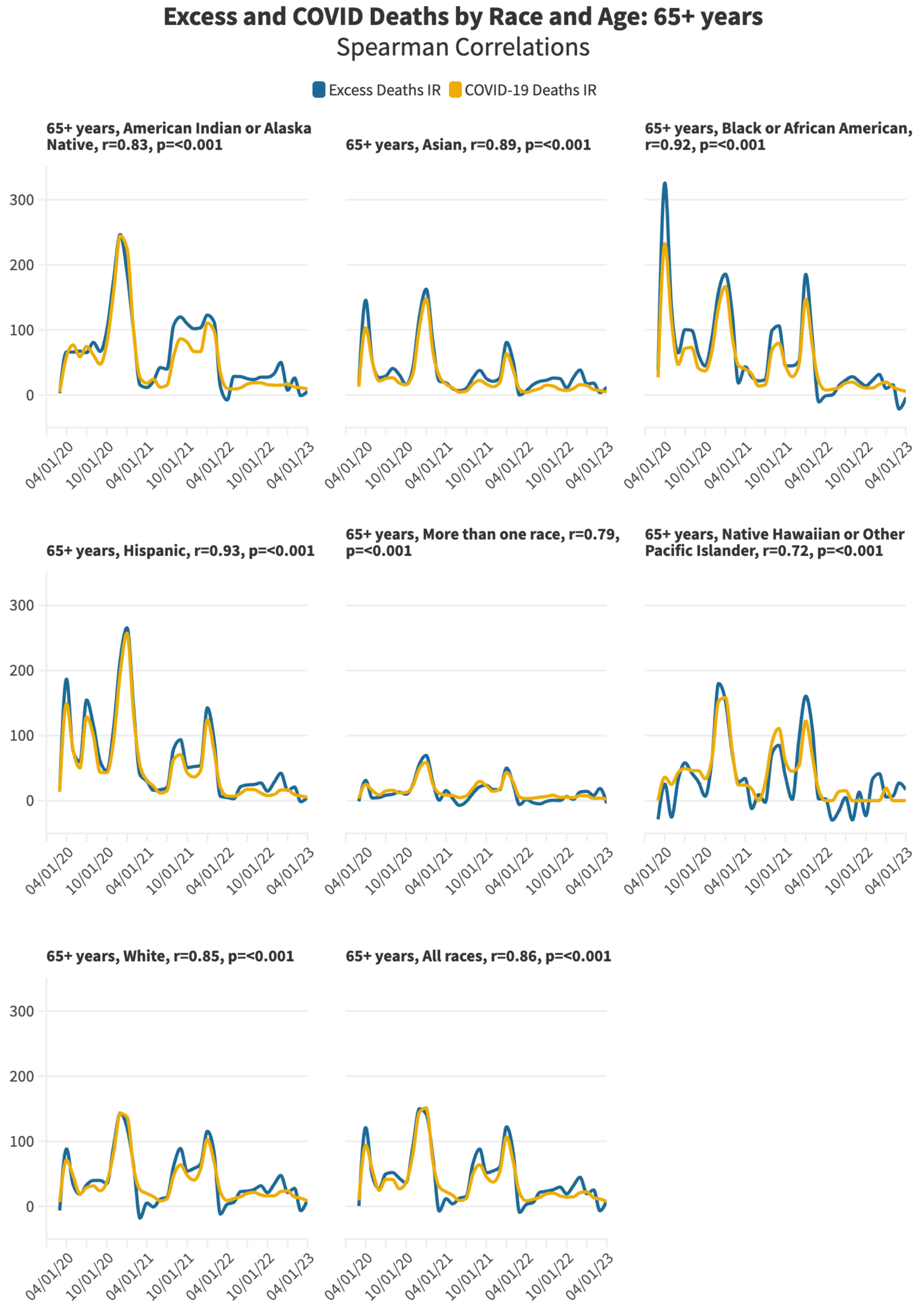


© 2024 Faust JS et al. *JAMA Network Open.*

**eTable 6.** Spearman correlation between all-cause excess mortality and Covid-19-specific mortality (multiple cause of death) by race/ethnicity and age group, with corresponding p values.

## Correlation between All-Cause Excess and COVID Mortality

| Age Group | Race | Spearman Correlation | pvalue |
|---|---|---|---|
| All ages | All races | 0.87 | <0.001 |
| All ages | American Indian or Alaska Native | 0.90 | <0.001 |
| All ages | Asian | 0.89 | <0.001 |
| All ages | Black or African American | 0.95 | <0.001 |
| All ages | Hispanic | 0.95 | <0.001 |
| All ages | More than one race | 0.81 | <0.001 |
| All ages | Native Hawaiian or Other Pacific Islander | 0.83 | <0.001 |
| All ages | White | 0.82 | <0.001 |
| <25 years | All races | 0.35 | 0.03 |
| 25-64 years | All races | 0.93 | <0.001 |
| 25-64 years | American Indian or Alaska Native | 0.91 | <0.001 |
| 25-64 years | Asian | 0.89 | <0.001 |
| 25-64 years | Black or African American | 0.94 | <0.001 |
| 25-64 years | Hispanic | 0.96 | <0.001 |
| 25-64 years | More than one race | 0.73 | <0.001 |
| 25-64 years | Native Hawaiian or Other Pacific Islander | 0.81 | <0.001 |
| 25-64 years | White | 0.87 | <0.001 |
| 65+ years | All races | 0.86 | <0.001 |
| 65+ years | American Indian or Alaska Native | 0.83 | <0.001 |
| 65+ years | Asian | 0.86 | <0.001 |
| 65+ years | Black or African American | 0.92 | <0.001 |
| 65+ years | Hispanic | 0.93 | <0.001 |
| 65+ years | More than one race | 0.79 | <0.001 |
| 65+ years | Native Hawaiian or Other Pacific Islander | 0.71 | <0.001 |
| 65+ years | White | 0.84 | <0.001 |

© 2024 Faust JS et al. *JAMA Network Open.*

**eTable 7.** Covid-19-Specific and All-Cause Excess Mortality Per 100,000 Persons by Race/Ethnicity, Ages <25 Years, With Spearman Correlations (When Possible)

| Age Group | Race | Cause of Death | Excess IR | Spearman to COVID Mortality | P value |
|---|---|---|---|---|---|
| All ages | White | A00-B99: Certain infectious and parasitic diseases | 3.06 | 0.19 | 0.25 |
| All ages | White | C00-D48: Neoplasms | 0.79 | 0.29 | 0.08 |
| All ages | White | D50-D89: Diseases of the blood and blood-forming organs and certain disorders involving the immune mechanism | 0.92 | 0.33 | 0.04 |
| All ages | White | E00-E88: Endocrine, nutritional and metabolic diseases | 35.28 | 0.37 | 0.02 |
| All ages | White | F01-F99: Mental and behavioural disorders | 6.16 | 0.62 | <0.001 |
| All ages | White | G00-G98: Diseases of the nervous system | 36.69 | 0.45 | 0.01 |
| All ages | White | I00-I99: Diseases of the circulatory system | 54.08 | 0.46 | 0 |
| All ages | White | J00-J98: Diseases of the respiratory system | -22.91 | 0.13 | 0.42 |
| All ages | White | K00-K92: Diseases of the digestive system | 20.14 | 0.42 | 0.01 |
| All ages | White | L00-L98: Diseases of the skin and subcutaneous tissue | 1.44 | -0.29 | 0.08 |
| All ages | White | M00-M99: Diseases of the musculoskeletal system and connective tissue | 0.57 | -0.05 | 0.78 |
| All ages | White | N00-N98: Diseases of the genitourinary system | 5.50 | 0.39 | 0.02 |
| All ages | White | O00-O99: Pregnancy, childbirth and the puerperium | 0.21 | 0.38 | 0.02 |
| All ages | White | P00-P96: Certain conditions originating in the perinatal period | -0.02 | 0.02 | 0.91 |
| All ages | White | Q00-Q99: Congenital malformations, deformations and chromosomal abnormalities | -0.07 | -0.38 | 0.02 |
| All ages | White | U00-U99: Codes for special purposes | 322.19 | 1.00 | <0.001 |
| All ages | White | V01-Y89: External causes of morbidity and mortality | 7.29 | 0.19 | 0.26 |

© 2024 Faust JS et al. *JAMA Network Open.*

| Age Group | Race | Cause of Death | Excess IR | Spearman to COVID Mortality | P value |
|---|---|---|---|---|---|
| All ages | Asian | A00-B99: Certain infectious and parasitic diseases | 5.06 | 0.02 | 0.89 |
| All ages | Asian | C00-D48: Neoplasms | 19.38 | -0.45 | 0.01 |
| All ages | Asian | E00-E88: Endocrine, nutritional and metabolic diseases | 17.47 | 0.02 | 0.89 |
| All ages | Asian | F01-F99: Mental and behavioural disorders | 0.94 | 0.01 | 0.97 |
| All ages | Asian | G00-G98: Diseases of the nervous system | 17.21 | -0.14 | 0.39 |
| All ages | Asian | I00-I99: Diseases of the circulatory system | 60.01 | 0.17 | 0.31 |
| All ages | Asian | J00-J98: Diseases of the respiratory system | 5.42 | 0.21 | 0.21 |
| All ages | Asian | K00-K92: Diseases of the digestive system | 5.66 | -0.16 | 0.33 |
| All ages | Asian | M00-M99: Diseases of the musculoskeletal system and connective tissue | 0.15 | 0.02 | 0.92 |
| All ages | Asian | N00-N98: Diseases of the genitourinary system | 2.80 | -0.20 | 0.23 |
| All ages | Asian | P00-P96: Certain conditions originating in the perinatal period | -0.63 | -0.25 | 0.12 |
| All ages | Asian | U00-U99: Codes for special purposes | 165.64 | 1.00 | <0.001 |
| All ages | Asian | V01-Y89: External causes of morbidity and mortality | 2.29 | -0.18 | 0.28 |
| All ages | Black or African American | A00-B99: Certain infectious and parasitic diseases | 5.42 | 0.35 | 0.03 |
| All ages | Black or African American | C00-D48: Neoplasms | -12.24 | -0.02 | 0.9 |
| All ages | Black or African American | D50-D89: Diseases of the blood and blood-forming organs and certain disorders involving the immune mechanism | 1.28 | 0.42 | 0.01 |
| All ages | Black or African American | E00-E88: Endocrine, nutritional and metabolic diseases | 34.52 | 0.68 | <0.001 |
| All ages | Black or African American | F01-F99: Mental and behavioural disorders | 6.93 | 0.73 | <0.001 |
| All ages | Black or African American | G00-G98: Diseases of the nervous system | 25.02 | 0.48 | 0 |
| All ages | Black or African American | I00-I99: Diseases of the circulatory system | 94.14 | 0.70 | <0.001 |
| All ages | Black or African American | J00-J98: Diseases of the respiratory system | 4.42 | 0.24 | 0.15 |
| All ages | Black or African American | K00-K92: Diseases of the digestive system | 14.62 | 0.30 | 0.07 |
| All ages | Black or African American | L00-L98: Diseases of the skin and subcutaneous tissue | 2.14 | -0.45 | 0 |
| All ages | Black or African American | M00-M99: Diseases of the musculoskeletal system and connective tissue | 2.02 | 0.16 | 0.33 |
| All ages | Black or African American | N00-N98: Diseases of the genitourinary system | 6.32 | 0.38 | 0.02 |
| All ages | Black or African American | O00-O99: Pregnancy, childbirth and the puerperium | 0.86 | 0.53 | <0.001 |
| All ages | Black or African American | P00-P96: Certain conditions originating in the perinatal period | -3.38 | 0.19 | 0.24 |
| All ages | Black or African American | Q00-Q99: Congenital malformations, deformations and chromosomal abnormalities | -0.69 | -0.34 | 0.04 |
| All ages | Black or African American | U00-U99: Codes for special purposes | 329.63 | 1.00 | <0.001 |
| All ages | Black or African American | V01-Y89: External causes of morbidity and mortality | 66.12 | 0.00 | 0.98 |
| All ages | Hispanic | A00-B99: Certain infectious and parasitic diseases | 3.51 | 0.14 | 0.4 |
| All ages | Hispanic | C00-D48: Neoplasms | 11.87 | -0.63 | <0.001 |
| All ages | Hispanic | D50-D89: Diseases of the blood and blood-forming organs and certain disorders involving the immune mechanism | 0.60 | -0.01 | 0.97 |
| All ages | Hispanic | E00-E88: Endocrine, nutritional and metabolic diseases | 19.12 | 0.12 | 0.47 |
| All ages | Hispanic | F01-F99: Mental and behavioural disorders | 6.31 | 0.13 | 0.45 |
| All ages | Hispanic | G00-G98: Diseases of the nervous system | 16.54 | -0.04 | 0.81 |
| All ages | Hispanic | I00-I99: Diseases of the circulatory system | 42.49 | 0.30 | 0.07 |
| All ages | Hispanic | J00-J98: Diseases of the respiratory system | 1.79 | -0.23 | 0.16 |
| All ages | Hispanic | K00-K92: Diseases of the digestive system | 14.74 | -0.15 | 0.36 |
| All ages | Hispanic | L00-L98: Diseases of the skin and subcutaneous tissue | 0.62 | -0.38 | 0.02 |
| All ages | Hispanic | M00-M99: Diseases of the musculoskeletal system and connective tissue | 1.04 | -0.25 | 0.13 |
| All ages | Hispanic | N00-N98: Diseases of the genitourinary system | 4.86 | -0.03 | 0.85 |
| All ages | Hispanic | P00-P96: Certain conditions originating in the perinatal period | -0.18 | -0.60 | <0.001 |
| All ages | Hispanic | Q00-Q99: Congenital malformations, deformations and chromosomal abnormalities | 0.17 | -0.37 | 0.02 |
| All ages | Hispanic | U00-U99: Codes for special purposes | 256.77 | 1.00 | <0.001 |
| All ages | Hispanic | V01-Y89: External causes of morbidity and mortality | 28.57 | -0.20 | 0.23 |
| All ages | More than one race | C00-D48: Neoplasms | 9.65 | -0.14 | 0.39 |
| All ages | More than one race | E00-E88: Endocrine, nutritional and metabolic diseases | 8.59 | 0.18 | 0.27 |
| All ages | More than one race | F01-F99: Mental and behavioural disorders | 4.12 | 0.27 | 0.1 |
| All ages | More than one race | G00-G98: Diseases of the nervous system | 5.39 | 0.08 | 0.64 |
| All ages | More than one race | I00-I99: Diseases of the circulatory system | 25.40 | -0.08 | 0.62 |
| All ages | More than one race | J00-J98: Diseases of the respiratory system | 1.44 | -0.29 | 0.08 |
| All ages | More than one race | K00-K92: Diseases of the digestive system | 6.81 | 0.16 | 0.33 |
| All ages | More than one race | P00-P96: Certain conditions originating in the perinatal period | 0.48 | -0.21 | 0.2 |
| All ages | More than one race | U00-U99: Codes for special purposes | 50.81 | 1.00 | <0.001 |
| All ages | More than one race | V01-Y89: External causes of morbidity and mortality | 27.01 | -0.12 | 0.47 |
| All ages | Native Hawaiian or Other Pacific Islander | C00-D48: Neoplasms | 2.10 | -0.33 | 0.04 |
| All ages | Native Hawaiian or Other Pacific Islander | I00-I99: Diseases of the circulatory system | 85.85 | 0.11 | 0.52 |
| All ages | Native Hawaiian or Other Pacific Islander | U00-U99: Codes for special purposes | 323.00 | 1.00 | <0.001 |
| All ages | Native Hawaiian or Other Pacific Islander | V01-Y89: External causes of morbidity and mortality | 53.33 | 0.17 | 0.3 |



© 2024 Faust JS et al. *JAMA Network Open.*

| Age Group | Race | Cause of Death | Excess IR | Spearman to COVID Mortality | P value |
|---|---|---|---|---|---|
| 65+ years | Black or African American | A00-B99: Certain infectious and parasitic diseases | 53.05 | 0.38 | 0.02 |
| 65+ years | Black or African American | C00-D48: Neoplasms | 90.01 | -0.38 | 0.02 |
| 65+ years | Black or African American | D50-D89: Diseases of the blood and blood-forming organs and certain disorders involving the immune mechanism | 7.74 | 0.14 | 0.41 |
| 65+ years | Black or African American | E00-E88: Endocrine, nutritional and metabolic diseases | 223.65 | 0.39 | 0.02 |
| 65+ years | Black or African American | F01-F99: Mental and behavioural disorders | 44.45 | 0.78 | <0.001 |
| 65+ years | Black or African American | G00-G98: Diseases of the nervous system | 186.13 | 0.39 | 0.02 |
| 65+ years | Black or African American | I00-I99: Diseases of the circulatory system | 534.93 | 0.34 | 0.03 |
| 65+ years | Black or African American | J00-J98: Diseases of the respiratory system | 57.06 | 0.26 | 0.11 |
| 65+ years | Black or African American | K00-K92: Diseases of the digestive system | 59.24 | -0.23 | 0.16 |
| 65+ years | Black or African American | L00-L98: Diseases of the skin and subcutaneous tissue | 12.69 | -0.34 | 0.04 |
| 65+ years | Black or African American | M00-M99: Diseases of the musculoskeletal system and connective tissue | 18.47 | -0.18 | 0.29 |
| 65+ years | Black or African American | N00-N98: Diseases of the genitourinary system | 53.74 | 0.27 | 0.11 |
| 65+ years | Black or African American | U00-U99: Codes for special purposes | 1,791.48 | 1.00 | <0.001 |
| 65+ years | Black or African American | V01-Y89: External causes of morbidity and mortality | 50.74 | 0.05 | 0.77 |
| 65+ years | Hispanic | A00-B99: Certain infectious and parasitic diseases | 35.49 | -0.08 | 0.65 |
| 65+ years | Hispanic | C00-D48: Neoplasms | 92.32 | -0.68 | <0.001 |
| 65+ years | Hispanic | D50-D89: Diseases of the blood and blood-forming organs and certain disorders involving the immune mechanism | 3.12 | 0.09 | 0.57 |
| 65+ years | Hispanic | E00-E88: Endocrine, nutritional and metabolic diseases | 124.25 | 0.26 | 0.12 |
| 65+ years | Hispanic | F01-F99: Mental and behavioural disorders | 19.94 | 0.09 | 0.57 |
| 65+ years | Hispanic | G00-G98: Diseases of the nervous system | 175.01 | 0.10 | 0.57 |
| 65+ years | Hispanic | I00-I99: Diseases of the circulatory system | 244.87 | 0.22 | 0.18 |
| 65+ years | Hispanic | J00-J98: Diseases of the respiratory system | 4.37 | -0.16 | 0.35 |
| 65+ years | Hispanic | K00-K92: Diseases of the digestive system | 52.31 | -0.42 | 0.01 |
| 65+ years | Hispanic | M00-M99: Diseases of the musculoskeletal system and connective tissue | 6.33 | -0.44 | 0.01 |
| 65+ years | Hispanic | N00-N98: Diseases of the genitourinary system | 23.86 | -0.12 | 0.49 |
| 65+ years | Hispanic | U00-U99: Codes for special purposes | 1,922.73 | 1.00 | <0.001 |
| 65+ years | Hispanic | V01-Y89: External causes of morbidity and mortality | 5.32 | 0.11 | 0.52 |
| 65+ years | More than one race | C00-D48: Neoplasms | 117.46 | -0.29 | 0.07 |
| 65+ years | More than one race | E00-E88: Endocrine, nutritional and metabolic diseases | 75.37 | 0.02 | 0.88 |
| 65+ years | More than one race | F01-F99: Mental and behavioural disorders | 21.46 | 0.08 | 0.63 |
| 65+ years | More than one race | G00-G98: Diseases of the nervous system | 81.73 | -0.07 | 0.67 |
| 65+ years | More than one race | I00-I99: Diseases of the circulatory system | 287.28 | -0.15 | 0.38 |
| 65+ years | More than one race | J00-J98: Diseases of the respiratory system | 17.96 | -0.35 | 0.03 |
| 65+ years | More than one race | U00-U99: Codes for special purposes | 531.58 | 1.00 | <0.001 |
| 65+ years | White | A00-B99: Certain infectious and parasitic diseases | 13.60 | -0.06 | 0.71 |
| 65+ years | White | C00-D48: Neoplasms | 39.26 | -0.05 | 0.79 |
| 65+ years | White | D50-D89: Diseases of the blood and blood-forming organs and certain disorders involving the immune mechanism | 2.73 | 0.29 | 0.08 |
| 65+ years | White | E00-E88: Endocrine, nutritional and metabolic diseases | 137.34 | 0.22 | 0.19 |
| 65+ years | White | F01-F99: Mental and behavioural disorders | 12.98 | 0.52 | <0.001 |
| 65+ years | White | G00-G98: Diseases of the nervous system | 167.28 | 0.36 | 0.03 |
| 65+ years | White | I00-I99: Diseases of the circulatory system | 183.77 | 0.34 | 0.04 |
| 65+ years | White | J00-J98: Diseases of the respiratory system | -109.77 | 0.09 | 0.6 |
| 65+ years | White | K00-K92: Diseases of the digestive system | 49.61 | 0.03 | 0.88 |
| 65+ years | White | L00-L98: Diseases of the skin and subcutaneous tissue | 5.25 | -0.26 | 0.12 |
| 65+ years | White | M00-M99: Diseases of the musculoskeletal system and connective tissue | 6.76 | 0.04 | 0.8 |
| 65+ years | White | N00-N98: Diseases of the genitourinary system | 21.44 | 0.22 | 0.19 |
| 65+ years | White | Q00-Q99: Congenital malformations, deformations and chromosomal abnormalities | 1.28 | -0.21 | 0.2 |
| 65+ years | White | U00-U99: Codes for special purposes | 1,256.10 | 1.00 | <0.001 |
| 65+ years | White | V01-Y89: External causes of morbidity and mortality | -4.54 | 0.17 | 0.32 |
| All ages | American Indian or Alaska Native | A00-B99: Certain infectious and parasitic diseases | 11.67 | 0.01 | 0.96 |
| All ages | American Indian or Alaska Native | C00-D48: Neoplasms | 18.65 | -0.11 | 0.51 |
| All ages | American Indian or Alaska Native | E00-E88: Endocrine, nutritional and metabolic diseases | 49.24 | 0.50 | 0 |
| All ages | American Indian or Alaska Native | F01-F99: Mental and behavioural disorders | 24.19 | 0.21 | 0.2 |
| All ages | American Indian or Alaska Native | G00-G98: Diseases of the nervous system | 20.94 | 0.33 | 0.05 |
| All ages | American Indian or Alaska Native | I00-I99: Diseases of the circulatory system | 81.14 | 0.42 | 0.01 |
| All ages | American Indian or Alaska Native | J00-J98: Diseases of the respiratory system | 3.53 | -0.03 | 0.87 |



© 2024 Faust JS et al. *JAMA Network Open.*

| Age Group | Race | Cause of Death | Excess IR | Spearman to COVID Mortality | P value |
| --- | --- | --- | --- | --- | --- |
| 25-64 years | Black or African American | K00-K92: Diseases of the digestive system | 14.37 | 0.60 | <0.001 |
| 25-64 years | Black or African American | M00-M99: Diseases of the musculoskeletal system and connective tissue | 0.89 | 0.43 | 0.01 |
| 25-64 years | Black or African American | N00-N98: Diseases of the genitourinary system | 5.15 | 0.47 | 0 |
| 25-64 years | Black or African American | Q00-Q99: Congenital malformations, deformations and chromosomal abnormalities | -0.12 | -0.30 | 0.07 |
| 25-64 years | Black or African American | U00-U99: Codes for special purposes | 214.47 | 1.00 | <0.001 |
| 25-64 years | Black or African American | V01-Y89: External causes of morbidity and mortality | 117.92 | 0.12 | 0.46 |
| 25-64 years | Hispanic | A00-B99: Certain infectious and parasitic diseases | 1.80 | 0.30 | 0.06 |
| 25-64 years | Hispanic | C00-D48: Neoplasms | 3.71 | -0.39 | 0.02 |
| 25-64 years | Hispanic | D50-D89: Diseases of the blood and blood-forming organs and certain disorders involving the immune mechanism | 0.38 | -0.07 | 0.7 |
| 25-64 years | Hispanic | E00-E88: Endocrine, nutritional and metabolic diseases | 11.99 | 0.37 | 0.02 |
| 25-64 years | Hispanic | F01-F99: Mental and behavioural disorders | 6.47 | -0.02 | 0.92 |
| 25-64 years | Hispanic | G00-G98: Diseases of the nervous system | 3.00 | -0.09 | 0.57 |
| 25-64 years | Hispanic | I00-I99: Diseases of the circulatory system | 27.03 | 0.46 | 0 |
| 25-64 years | Hispanic | J00-J98: Diseases of the respiratory system | 0.35 | 0.13 | 0.44 |
| 25-64 years | Hispanic | K00-K92: Diseases of the digestive system | 19.86 | 0.37 | 0.02 |
| 25-64 years | Hispanic | M00-M99: Diseases of the musculoskeletal system and connective tissue | 0.66 | 0.04 | 0.8 |
| 25-64 years | Hispanic | N00-N98: Diseases of the genitourinary system | 2.77 | -0.01 | 0.97 |
| 25-64 years | Hispanic | U00-U99: Codes for special purposes | 202.97 | 1.00 | <0.001 |
| 25-64 years | Hispanic | V01-Y89: External causes of morbidity and mortality | 43.59 | -0.15 | 0.37 |
| 25-64 years | More than one race | C00-D48: Neoplasms | 7.82 | 0.09 | 0.58 |
| 25-64 years | More than one race | I00-I99: Diseases of the circulatory system | 22.89 | 0.10 | 0.56 |
| 25-64 years | More than one race | U00-U99: Codes for special purposes | 50.06 | 1.00 | <0.001 |
| 25-64 years | More than one race | V01-Y89: External causes of morbidity and mortality | 57.14 | -0.07 | 0.69 |
| 25-64 years | White | A00-B99: Certain infectious and parasitic diseases | 0.97 | 0.54 | <0.001 |
| 25-64 years | White | C00-D48: Neoplasms | -20.81 | 0.52 | <0.001 |
| 25-64 years | White | D50-D89: Diseases of the blood and blood-forming organs and certain disorders involving the immune mechanism | 0.52 | 0.06 | 0.74 |
| 25-64 years | White | E00-E88: Endocrine, nutritional and metabolic diseases | 10.78 | 0.71 | <0.001 |
| 25-64 years | White | F01-F99: Mental and behavioural disorders | 7.64 | 0.76 | <0.001 |
| 25-64 years | White | G00-G98: Diseases of the nervous system | 1.11 | 0.28 | 0.09 |
| 25-64 years | White | I00-I99: Diseases of the circulatory system | 19.42 | 0.80 | <0.001 |
| 25-64 years | White | J00-J98: Diseases of the respiratory system | -6.64 | 0.38 | 0.02 |
| 25-64 years | White | K00-K92: Diseases of the digestive system | 16.60 | 0.78 | <0.001 |
| 25-64 years | White | L00-L98: Diseases of the skin and subcutaneous tissue | 0.59 | -0.16 | 0.32 |
| 25-64 years | White | M00-M99: Diseases of the musculoskeletal system and connective tissue | 0.42 | 0.17 | 0.31 |
| 25-64 years | White | N00-N98: Diseases of the genitourinary system | 1.85 | 0.50 | 0 |
| 25-64 years | White | O00-O99: Pregnancy, childbirth and the puerperium | 0.41 | 0.44 | 0.01 |
| 25-64 years | White | Q00-Q99: Congenital malformations, deformations and chromosomal abnormalities | 0.41 | 0.03 | 0.84 |
| 25-64 years | White | U00-U99: Codes for special purposes | 114.50 | 1.00 | <0.001 |
| 25-64 years | White | V01-Y89: External causes of morbidity and mortality | 15.25 | 0.41 | 0.01 |
| 65+ years | American Indian or Alaska Native | C00-D48: Neoplasms | 133.62 | -0.22 | 0.17 |
| 65+ years | American Indian or Alaska Native | E00-E88: Endocrine, nutritional and metabolic diseases | 204.59 | 0.23 | 0.17 |
| 65+ years | American Indian or Alaska Native | F01-F99: Mental and behavioural disorders | 43.36 | 0.08 | 0.62 |
| 65+ years | American Indian or Alaska Native | G00-G98: Diseases of the nervous system | 128.58 | 0.28 | 0.09 |
| 65+ years | American Indian or Alaska Native | I00-I99: Diseases of the circulatory system | 369.97 | 0.13 | 0.43 |
| 65+ years | American Indian or Alaska Native | J00-J98: Diseases of the respiratory system | -84.74 | -0.11 | 0.49 |
| 65+ years | American Indian or Alaska Native | K00-K92: Diseases of the digestive system | 87.85 | 0.21 | 0.21 |
| 65+ years | American Indian or Alaska Native | N00-N98: Diseases of the genitourinary system | 39.05 | -0.05 | 0.79 |
| 65+ years | American Indian or Alaska Native | U00-U99: Codes for special purposes | 2,012.75 | 1.00 | <0.001 |
| 65+ years | American Indian or Alaska Native | V01-Y89: External causes of morbidity and mortality | 17.64 | 0.17 | 0.31 |
| 65+ years | Asian | A00-B99: Certain infectious and parasitic diseases | 31.67 | -0.07 | 0.7 |
| 65+ years | Asian | C00-D48: Neoplasms | 176.62 | -0.40 | 0.01 |
| 65+ years | Asian | E00-E88: Endocrine, nutritional and metabolic diseases | 151.74 | 0.11 | 0.49 |
| 65+ years | Asian | F01-F99: Mental and behavioural disorders | 34.20 | 0.22 | 0.19 |
| 65+ years | Asian | G00-G98: Diseases of the nervous system | 169.34 | -0.02 | 0.93 |
| 65+ years | Asian | I00-I99: Diseases of the circulatory system | 469.29 | 0.02 | 0.92 |
| 65+ years | Asian | J00-J98: Diseases of the respiratory system | 22.86 | 0.26 | 0.11 |
| 65+ years | Asian | K00-K92: Diseases of the digestive system | 39.17 | -0.13 | 0.44 |
| 65+ years | Asian | N00-N98: Diseases of the genitourinary system | 30.66 | -0.17 | 0.31 |
| 65+ years | Asian | U00-U99: Codes for special purposes | 995.11 | 1.00 | <0.001 |
| 65+ years | Asian | V01-Y89: External causes of morbidity and mortality | 5.83 | 0.16 | 0.35 |



© 2024 Faust JS et al. *JAMA Network Open.*

| Age Group | Race | Cause of Death | Excess IR | Spearman to COVID Mortality | P value |
|---|---|---|---|---|---|
| <25 years | American Indian or Alaska Native | U00-U99: Codes for special purposes | 0.00 | | |
| <25 years | American Indian or Alaska Native | V01-Y89: External causes of morbidity and mortality | 21.47 | | |
| <25 years | Asian | P00-P96: Certain conditions originating in the perinatal period | -2.15 | -0.03 | 0.86 |
| <25 years | Asian | U00-U99: Codes for special purposes | 0.25 | 1.00 | <0.001 |
| <25 years | Asian | V01-Y89: External causes of morbidity and mortality | -0.10 | 0.23 | 0.16 |
| <25 years | Black or African American | A00-B99: Certain infectious and parasitic diseases | -0.58 | 0.10 | 0.57 |
| <25 years | Black or African American | C00-D48: Neoplasms | -0.41 | 0.03 | 0.84 |
| <25 years | Black or African American | G00-G98: Diseases of the nervous system | 0.91 | -0.03 | 0.86 |
| <25 years | Black or African American | I00-I99: Diseases of the circulatory system | -0.22 | 0.22 | 0.19 |
| <25 years | Black or African American | J00-J98: Diseases of the respiratory system | -2.16 | 0.07 | 0.67 |
| <25 years | Black or African American | P00-P96: Certain conditions originating in the perinatal period | -9.21 | 0.05 | 0.76 |
| <25 years | Black or African American | Q00-Q99: Congenital malformations, deformations and chromosomal abnormalities | -2.27 | 0.03 | 0.87 |
| <25 years | Black or African American | U00-U99: Codes for special purposes | 4.94 | 1.00 | <0.001 |
| <25 years | Black or African American | V01-Y89: External causes of morbidity and mortality | 41.43 | 0.23 | 0.17 |
| <25 years | Hispanic | C00-D48: Neoplasms | -0.01 | 0.24 | 0.15 |
| <25 years | Hispanic | G00-G98: Diseases of the nervous system | 0.16 | -0.12 | 0.47 |
| <25 years | Hispanic | I00-I99: Diseases of the circulatory system | 0.37 | 0.28 | 0.09 |
| <25 years | Hispanic | J00-J98: Diseases of the respiratory system | -1.14 | -0.29 | 0.08 |
| <25 years | Hispanic | P00-P96: Certain conditions originating in the perinatal period | -0.59 | -0.39 | 0.01 |
| <25 years | Hispanic | Q00-Q99: Congenital malformations, deformations and chromosomal abnormalities | -0.14 | -0.18 | 0.28 |
| <25 years | Hispanic | U00-U99: Codes for special purposes | 3.61 | 1.00 | <0.001 |
| <25 years | Hispanic | V01-Y89: External causes of morbidity and mortality | 14.63 | 0.08 | 0.62 |
| <25 years | More than one race | P00-P96: Certain conditions originating in the perinatal period | 2.42 | | |
| <25 years | More than one race | U00-U99: Codes for special purposes | 0.00 | | |
| <25 years | More than one race | V01-Y89: External causes of morbidity and mortality | 8.40 | | |
| <25 years | White | A00-B99: Certain infectious and parasitic diseases | -0.36 | 0.06 | 0.73 |
| <25 years | White | C00-D48: Neoplasms | -0.24 | 0.01 | 0.95 |
| <25 years | White | E00-E88: Endocrine, nutritional and metabolic diseases | -0.20 | 0.36 | 0.02 |
| <25 years | White | G00-G98: Diseases of the nervous system | 0.77 | 0.22 | 0.19 |
| <25 years | White | I00-I99: Diseases of the circulatory system | -0.69 | -0.04 | 0.82 |
| <25 years | White | J00-J98: Diseases of the respiratory system | -1.04 | -0.33 | 0.04 |
| <25 years | White | P00-P96: Certain conditions originating in the perinatal period | -0.10 | -0.03 | 0.84 |
| <25 years | White | Q00-Q99: Congenital malformations, deformations and chromosomal abnormalities | -1.21 | 0.05 | 0.75 |
| <25 years | White | U00-U99: Codes for special purposes | 2.22 | 1.00 | <0.001 |
| <25 years | White | V01-Y89: External causes of morbidity and mortality | 3.00 | 0.07 | 0.66 |
| 25-64 years | American Indian or Alaska Native | C00-D48: Neoplasms | 2.09 | 0.20 | 0.23 |
| 25-64 years | American Indian or Alaska Native | E00-E88: Endocrine, nutritional and metabolic diseases | 42.60 | 0.49 | 0 |
| 25-64 years | American Indian or Alaska Native | F01-F99: Mental and behavioural disorders | 35.30 | 0.18 | 0.29 |
| 25-64 years | American Indian or Alaska Native | I00-I99: Diseases of the circulatory system | 55.62 | 0.58 | <0.001 |
| 25-64 years | American Indian or Alaska Native | J00-J98: Diseases of the respiratory system | 14.07 | 0.20 | 0.22 |
| 25-64 years | American Indian or Alaska Native | K00-K92: Diseases of the digestive system | 154.36 | 0.60 | <0.001 |
| 25-64 years | American Indian or Alaska Native | U00-U99: Codes for special purposes | 364.09 | 1.00 | <0.001 |
| 25-64 years | American Indian or Alaska Native | V01-Y89: External causes of morbidity and mortality | 144.02 | 0.12 | 0.47 |
| 25-64 years | Asian | A00-B99: Certain infectious and parasitic diseases | 2.35 | 0.15 | 0.35 |
| 25-64 years | Asian | C00-D48: Neoplasms | 4.23 | -0.05 | 0.75 |
| 25-64 years | Asian | E00-E88: Endocrine, nutritional and metabolic diseases | 3.74 | 0.08 | 0.65 |
| 25-64 years | Asian | G00-G98: Diseases of the nervous system | 1.37 | -0.02 | 0.89 |
| 25-64 years | Asian | I00-I99: Diseases of the circulatory system | 13.98 | 0.50 | 0 |
| 25-64 years | Asian | J00-J98: Diseases of the respiratory system | -0.65 | 0.14 | 0.4 |
| 25-64 years | Asian | K00-K92: Diseases of the digestive system | 4.98 | 0.17 | 0.31 |
| 25-64 years | Asian | U00-U99: Codes for special purposes | 63.04 | 1.00 | <0.001 |
| 25-64 years | Asian | V01-Y89: External causes of morbidity and mortality | 3.56 | -0.18 | 0.28 |
| 25-64 years | Black or African American | A00-B99: Certain infectious and parasitic diseases | -0.03 | 0.43 | 0.01 |
| 25-64 years | Black or African American | C00-D48: Neoplasms | -24.98 | 0.67 | <0.001 |
| 25-64 years | Black or African American | D50-D89: Diseases of the blood and blood-forming organs and certain disorders involving the immune mechanism | 1.06 | 0.54 | <0.001 |
| 25-64 years | Black or African American | E00-E88: Endocrine, nutritional and metabolic diseases | 29.52 | 0.87 | <0.001 |
| 25-64 years | Black or African American | F01-F99: Mental and behavioural disorders | 8.88 | 0.58 | <0.001 |
| 25-64 years | Black or African American | G00-G98: Diseases of the nervous system | 3.93 | 0.24 | 0.14 |
| 25-64 years | Black or African American | I00-I99: Diseases of the circulatory system | 59.90 | 0.89 | <0.001 |



© 2024 Faust JS et al. *JAMA Network Open.*

**eTable S8.** Relative risks by age and race/ethnicity, pre-pandemic and pandemic periods.

| | Pre-pandemic | Pandemic | Pre-pandemic | Pandemic | Pre-pandemic | Pandemic | Pre-pandemic | Pandemic |
|---|---|---|---|---|---|---|---|---|
| | <25 years | <25 years | 25-64 years | 25-64 years | 65+ years | 65+ years | All ages | All ages |
| American Indian or Alaska Native | 1.85 (1.80 - 1.90) | 2.16 (2.08 - 2.24) | 1.64 (1.62 - 1.66) | 2.10 (2.08 - 2.13) | 0.85 (0.84 - 0.86) | 0.85 (0.85 - 0.86) | 1.07 (1.06 - 1.08) | 1.22 (1.21 - 1.22) |
| Asian | 0.59 (0.58 - 0.61) | 0.57 (0.56 - 0.59) | 0.37 (0.37 - 0.38) | 0.38 (0.38 - 0.38) | 0.56 (0.56 - 0.56) | 0.57 (0.57 - 0.57) | 0.51 (0.51 - 0.51) | 0.52 (0.52 - 0.52) |
| Black or African American | 1.96 (1.94 - 1.97) | 2.31 (2.29 - 2.33) | 1.45 (1.45 - 1.46) | 1.58 (1.57 - 1.58) | 1.07 (1.07 - 1.07) | 1.11 (1.11 - 1.12) | 1.19 (1.19 - 1.19) | 1.26 (1.26 - 1.27) |
| Hispanic | 0.90 (0.89 - 0.90) | 1.04 (1.02 - 1.05) | 0.66 (0.66 - 0.67) | 0.80 (0.79 - 0.80) | 0.72 (0.71 - 0.72) | 0.79 (0.79 - 0.79) | 0.71 (0.71 - 0.71) | 0.80 (0.79 - 0.80) |
| More than one race | 0.67 (0.65 - 0.68) | 0.75 (0.73 - 0.77) | 0.52 (0.52 - 0.53) | 0.59 (0.58 - 0.60) | 0.41 (0.40 - 0.41) | 0.40 (0.40 - 0.41) | 0.44 (0.44 - 0.45) | 0.46 (0.46 - 0.46) |
| Native Hawaiian or Other Pacific Islander | 1.50 (1.41 - 1.60) | 1.79 (1.66 - 1.93) | 1.20 (1.17 - 1.23) | 1.50 (1.47 - 1.54) | 0.79 (0.77 - 0.80) | 0.78 (0.76 - 0.80) | 0.91 (0.90 - 0.92) | 1.00 (0.98 - 1.02) |
| White | 1.00 (0.99 - 1.01) | 1.00 (0.99 - 1.01) | 1.00 (1.00 - 1.00) | 1.00 (1.00 - 1.00) | 1.00 (1.00 - 1.00) | 1.00 (1.00 - 1.00) | 1.00 (1.00 - 1.00) | 1.00 (1.00 - 1.00) |

**eTable 9.** Relative risks by age and race/ethnicity, pandemic period (pre-vaccine and post-vaccine periods).

| | Pre-vaccine | Post-vaccine | Pre-vaccine | Post-vaccine | Pre-vaccine | Post-vaccine | Pre-vaccine | Post-vaccine |
|---|---|---|---|---|---|---|---|---|
| | <25 years | <25 years | 25-64 years | 25-64 years | 65+ years | 65+ years | All ages | All ages |
| American Indian or Alaska Native | 2.00 (1.89 - 2.13) | 2.25 (2.16 - 2.35) | 2.15 (2.11 - 2.18) | 2.08 (2.05 - 2.10) | 0.93 (0.92 - 0.95) | 0.82 (0.81 - 0.83) | 1.26 (1.25 - 1.27) | 1.19 (1.18 - 1.20) |
| Asian | 0.54 (0.52 - 0.57) | 0.59 (0.57 - 0.61) | 0.42 (0.41 - 0.42) | 0.36 (0.35 - 0.36) | 0.61 (0.61 - 0.62) | 0.55 (0.55 - 0.55) | 0.56 (0.56 - 0.56) | 0.50 (0.49 - 0.50) |
| Black or African American | 2.30 (2.26 - 2.34) | 2.32 (2.29 - 2.35) | 1.67 (1.66 - 1.67) | 1.52 (1.52 - 1.53) | 1.22 (1.22 - 1.23) | 1.07 (1.06 - 1.07) | 1.36 (1.35 - 1.36) | 1.21 (1.21 - 1.22) |
| Hispanic | 1.00 (0.98 - 1.02) | 1.06 (1.04 - 1.07) | 0.90 (0.89 - 0.90) | 0.74 (0.74 - 0.75) | 0.90 (0.90 - 0.91) | 0.74 (0.74 - 0.74) | 0.89 (0.89 - 0.90) | 0.74 (0.74 - 0.75) |
| More than one race | 0.72 (0.68 - 0.75) | 0.78 (0.75 - 0.80) | 0.58 (0.57 - 0.59) | 0.59 (0.58 - 0.60) | 0.40 (0.39 - 0.41) | 0.40 (0.40 - 0.41) | 0.46 (0.45 - 0.46) | 0.46 (0.46 - 0.47) |
| Native Hawaiian or Other Pacific Islander | 1.57 (1.37 - 1.79) | 1.93 (1.76 - 2.11) | 1.51 (1.46 - 1.58) | 1.49 (1.45 - 1.54) | 0.79 (0.76 - 0.83) | 0.78 (0.75 - 0.80) | 1.01 (0.98 - 1.03) | 1.00 (0.98 - 1.02) |
| White | 1.00 (0.98 - 1.02) | 1.00 (0.99 - 1.01) | 1.00 (1.00 - 1.00) | 1.00 (1.00 - 1.00) | 1.00 (1.00 - 1.00) | 1.00 (1.00 - 1.00) | 1.00 (1.00 - 1.00) | 1.00 (1.00 - 1.00) |

© 2024 Faust JS et al. *JAMA Network Open.*

**eTable 10.** Relative risks by age and race/ethnicity, by pandemic year, All ages.

| | March 2015-February 2016 | March 2016-February 2017 | March 2017-February 2018 | March 2018-February 2019 | March 2019-February 2020 | March 2020-February 2021 | March 2021-February 2022 | March 2022-April 2023 |
|---|---|---|---|---|---|---|---|---|
| | All ages | All ages | All ages | All ages | All ages | All ages | All ages | All ages |
| American Indian or Alaska Native | 1.09 (1.08 - 1.11) | 1.08 (1.07 - 1.10) | 1.07 (1.05 - 1.08) | 1.07 (1.05 - 1.08) | 1.05 (1.04 - 1.07) | 1.27 (1.26 - 1.29) | 1.26 (1.25 - 1.28) | 1.13 (1.12 - 1.15) |
| Asian | 0.52 (0.52 - 0.53) | 0.51 (0.51 - 0.52) | 0.51 (0.51 - 0.52) | 0.51 (0.51 - 0.51) | 0.50 (0.50 - 0.51) | 0.56 (0.56 - 0.57) | 0.49 (0.49 - 0.50) | 0.51 (0.50 - 0.51) |
| Black or African American | 1.19 (1.18 - 1.19) | 1.19 (1.18 - 1.19) | 1.19 (1.19 - 1.19) | 1.19 (1.19 - 1.20) | 1.20 (1.19 - 1.20) | 1.36 (1.36 - 1.37) | 1.26 (1.26 - 1.27) | 1.19 (1.19 - 1.19) |
| Hispanic | 0.72 (0.71 - 0.72) | 0.71 (0.71 - 0.71) | 0.71 (0.70 - 0.71) | 0.70 (0.70 - 0.70) | 0.71 (0.70 - 0.71) | 0.91 (0.91 - 0.91) | 0.78 (0.78 - 0.79) | 0.72 (0.72 - 0.72) |
| More than one race | 0.44 (0.43 - 0.44) | 0.44 (0.43 - 0.45) | 0.46 (0.45 - 0.47) | 0.45 (0.44 - 0.46) | 0.44 (0.43 - 0.45) | 0.46 (0.45 - 0.46) | 0.46 (0.45 - 0.47) | 0.46 (0.46 - 0.47) |
| Native Hawaiian or Other Pacific Islander | 0.90 (0.87 - 0.94) | 0.91 (0.88 - 0.94) | 0.91 (0.88 - 0.94) | 0.91 (0.88 - 0.94) | 0.92 (0.89 - 0.95) | 0.99 (0.97 - 1.02) | 1.07 (1.04 - 1.10) | 0.95 (0.92 - 0.97) |
| White | 1.00 (1.00 - 1.00) | 1.00 (1.00 - 1.00) | 1.00 (1.00 - 1.00) | 1.00 (1.00 - 1.00) | 1.00 (1.00 - 1.00) | 1.00 (1.00 - 1.00) | 1.00 (1.00 - 1.00) | 1.00 (1.00 - 1.00) |

**eTable 11.** Relative risks by age and race/ethnicity, by pandemic year, Ages <25 years.

| | March 2015-February 2016 | March 2016-February 2017 | March 2017-February 2018 | March 2018-February 2019 | March 2019-February 2020 | March 2020-February 2021 | March 2021-February 2022 | March 2022-April 2023 |
|---|---|---|---|---|---|---|---|---|
| | <25 years | <25 years | <25 years | <25 years | <25 years | <25 years | <25 years | <25 years |
| American Indian or Alaska Native | 1.83 (1.71 - 1.95) | 1.83 (1.72 - 1.95) | 1.86 (1.74 - 1.99) | 1.82 (1.70 - 1.95) | 1.95 (1.82 - 2.08) | 2.03 (1.91 - 2.17) | 2.16 (2.03 - 2.30) | 2.27 (2.14 - 2.40) |
| Asian | 0.60 (0.57 - 0.63) | 0.59 (0.56 - 0.62) | 0.59 (0.57 - 0.62) | 0.60 (0.57 - 0.63) | 0.58 (0.56 - 0.61) | 0.53 (0.51 - 0.56) | 0.58 (0.56 - 0.61) | 0.60 (0.57 - 0.62) |
| Black or African American | 1.90 (1.87 - 1.94) | 1.92 (1.88 - 1.96) | 1.91 (1.87 - 1.94) | 1.97 (1.93 - 2.01) | 2.09 (2.05 - 2.13) | 2.30 (2.25 - 2.34) | 2.33 (2.29 - 2.38) | 2.30 (2.26 - 2.34) |
| Hispanic | 0.88 (0.86 - 0.90) | 0.87 (0.85 - 0.89) | 0.88 (0.87 - 0.90) | 0.89 (0.87 - 0.91) | 0.97 (0.95 - 0.99) | 1.00 (0.98 - 1.03) | 1.03 (1.01 - 1.05) | 1.07 (1.05 - 1.09) |
| More than one race | 0.60 (0.57 - 0.63) | 0.60 (0.57 - 0.64) | 0.70 (0.67 - 0.74) | 0.69 (0.66 - 0.73) | 0.75 (0.71 - 0.78) | 0.72 (0.68 - 0.75) | 0.75 (0.71 - 0.78) | 0.79 (0.76 - 0.82) |
| Native Hawaiian or Other Pacific Islander | 1.23 (1.05 - 1.45) | 1.51 (1.31 - 1.74) | 1.45 (1.25 - 1.68) | 1.63 (1.41 - 1.88) | 1.79 (1.56 - 2.06) | 1.51 (1.30 - 1.75) | 1.89 (1.66 - 2.15) | 1.97 (1.75 - 2.22) |
| White | 1.00 (0.98 - 1.02) | 1.00 (0.98 - 1.02) | 1.00 (0.98 - 1.02) | 1.00 (0.98 - 1.02) | 1.00 (0.98 - 1.02) | 1.00 (0.98 - 1.02) | 1.00 (0.98 - 1.02) | 1.00 (0.98 - 1.02) |

**eTable 12.** Relative risks by age and race/ethnicity, by pandemic year, Ages 25-64 years.

| | March 2015-February 2016 | March 2016-February 2017 | March 2017-February 2018 | March 2018-February 2019 | March 2019-February 2020 | March 2020-February 2021 | March 2021-February 2022 | March 2022-April 2023 |
|---|---|---|---|---|---|---|---|---|
| | 25-64 years | 25-64 years | 25-64 years | 25-64 years | 25-64 years | 25-64 years | 25-64 years | 25-64 years |
| American Indian or Alaska Native | 1.63 (1.59 - 1.67) | 1.60 (1.57 - 1.64) | 1.62 (1.59 - 1.66) | 1.68 (1.64 - 1.72) | 1.68 (1.64 - 1.71) | 2.18 (2.14 - 2.22) | 2.13 (2.10 - 2.17) | 2.01 (1.98 - 2.05) |
| Asian | 0.38 (0.37 - 0.39) | 0.37 (0.37 - 0.38) | 0.37 (0.36 - 0.38) | 0.37 (0.37 - 0.38) | 0.37 (0.36 - 0.38) | 0.42 (0.41 - 0.43) | 0.36 (0.35 - 0.36) | 0.37 (0.36 - 0.37) |
| Black or African American | 1.44 (1.43 - 1.45) | 1.44 (1.43 - 1.45) | 1.45 (1.44 - 1.46) | 1.46 (1.45 - 1.47) | 1.47 (1.46 - 1.48) | 1.67 (1.67 - 1.68) | 1.55 (1.55 - 1.56) | 1.51 (1.50 - 1.52) |
| Hispanic | 0.67 (0.66 - 0.67) | 0.66 (0.65 - 0.66) | 0.66 (0.66 - 0.67) | 0.66 (0.66 - 0.67) | 0.67 (0.67 - 0.68) | 0.92 (0.91 - 0.92) | 0.79 (0.78 - 0.79) | 0.72 (0.71 - 0.72) |
| More than one race | 0.50 (0.48 - 0.52) | 0.50 (0.48 - 0.51) | 0.55 (0.53 - 0.56) | 0.54 (0.52 - 0.55) | 0.54 (0.53 - 0.56) | 0.58 (0.57 - 0.60) | 0.58 (0.56 - 0.59) | 0.61 (0.59 - 0.62) |
| Native Hawaiian or Other Pacific Islander | 1.19 (1.13 - 1.26) | 1.13 (1.07 - 1.19) | 1.20 (1.14 - 1.26) | 1.22 (1.16 - 1.29) | 1.24 (1.18 - 1.31) | 1.52 (1.45 - 1.58) | 1.61 (1.55 - 1.67) | 1.40 (1.35 - 1.46) |
| White | 1.00 (1.00 - 1.00) | 1.00 (1.00 - 1.00) | 1.00 (1.00 - 1.00) | 1.00 (1.00 - 1.00) | 1.00 (1.00 - 1.00) | 1.00 (1.00 - 1.00) | 1.00 (1.00 - 1.00) | 1.00 (1.00 - 1.00) |

© 2024 Faust JS et al. *JAMA Network Open.*

**eTable 13.** Relative risks by age and race/ethnicity, by pandemic year, Ages ≥65 years.

| | March 2015-February 2016 | March 2016-February 2017 | March 2017-February 2018 | March 2018-February 2019 | March 2019-February 2020 | March 2020-February 2021 | March 2021-February 2022 | March 2022-April 2023 |
|---|---|---|---|---|---|---|---|---|
| | 65+ years | 65+ years | 65+ years | 65+ years | 65+ years | 65+ years | 65+ years | 65+ years |
| American Indian or Alaska Native | 0.88 (0.86 - 0.90) | 0.88 (0.86 - 0.90) | 0.84 (0.83 - 0.86) | 0.83 (0.81 - 0.84) | 0.80 (0.79 - 0.82) | 0.93 (0.92 - 0.95) | 0.88 (0.86 - 0.89) | 0.77 (0.76 - 0.79) |
| Asian | 0.57 (0.56 - 0.57) | 0.56 (0.55 - 0.56) | 0.56 (0.55 - 0.56) | 0.55 (0.55 - 0.56) | 0.55 (0.54 - 0.55) | 0.61 (0.61 - 0.62) | 0.55 (0.54 - 0.55) | 0.55 (0.55 - 0.56) |
| Black or African American | 1.07 (1.07 - 1.08) | 1.07 (1.07 - 1.08) | 1.07 (1.07 - 1.08) | 1.07 (1.07 - 1.08) | 1.07 (1.06 - 1.07) | 1.22 (1.22 - 1.23) | 1.11 (1.10 - 1.11) | 1.03 (1.03 - 1.04) |
| Hispanic | 0.73 (0.72 - 0.73) | 0.72 (0.72 - 0.72) | 0.72 (0.71 - 0.72) | 0.71 (0.70 - 0.71) | 0.71 (0.71 - 0.71) | 0.90 (0.90 - 0.91) | 0.77 (0.77 - 0.78) | 0.71 (0.71 - 0.71) |
| More than one race | 0.41 (0.40 - 0.42) | 0.41 (0.40 - 0.42) | 0.42 (0.41 - 0.43) | 0.41 (0.40 - 0.42) | 0.40 (0.39 - 0.41) | 0.40 (0.39 - 0.41) | 0.40 (0.39 - 0.41) | 0.40 (0.39 - 0.41) |
| Native Hawaiian or Other Pacific Islander | 0.79 (0.75 - 0.83) | 0.80 (0.77 - 0.85) | 0.79 (0.75 - 0.82) | 0.77 (0.73 - 0.81) | 0.77 (0.74 - 0.81) | 0.79 (0.76 - 0.83) | 0.82 (0.78 - 0.85) | 0.74 (0.71 - 0.77) |
| White | 1.00 (1.00 - 1.00) | 1.00 (1.00 - 1.00) | 1.00 (1.00 - 1.00) | 1.00 (1.00 - 1.00) | 1.00 (1.00 - 1.00) | 1.00 (1.00 - 1.00) | 1.00 (1.00 - 1.00) | 1.00 (1.00 - 1.00) |

© 2024 Faust JS et al. *JAMA Network Open.*
