# Supplementary material for: Racial and Ethnic Disparities in Age-Specific All-Cause Mortality During the COVID-19 Pandemic
Source: JAMA Netw Open. 2024 Oct 11;7(10):e2438918. doi: 10.1001/jamanetworkopen.2024.38918 (PMC11581672; doi:10.1001/jamanetworkopen.2024.38918)
Supplement: Supplement 2. — Data Sharing Statement [file jamanetwopen-e2438918-s002.pdf]

Data Sharing Statement

Faust. Racial and Ethnic Disparities in Age-Specific All-Cause Mortality During the COVID-19 Pandemic. *JAMA Netw Open*. Published October 11, 2024.
doi:10.1001/jamanetworkopen.2024.38918

Data
Data available: No

Additional Information
Explanation for why data not available: The data are public already
